# An aluminum shield enables the amphipod *Hirondellea gigas* to inhabit deep-sea environments

**DOI:** 10.1371/journal.pone.0206710

**Published:** 2019-04-04

**Authors:** Hideki Kobayashi, Hirokazu Shimoshige, Yoshikata Nakajima, Wataru Arai, Hideto Takami

**Affiliations:** 1 Japan Agency for Marine-Earth Science and Technology (JAMSTEC) Natsushima, Yokosuka, Japan; 2 Bio-nano Electronics Research Centre, Toyo University, Kujirai, Kawagoe, Saitama, Japan; Centre National de la Recherche Scientifique, FRANCE

## Abstract

The amphipod *Hirondellea gigas* inhabits the deepest regions of the oceans in extreme high-pressure conditions. However, the mechanisms by which this amphipod adapts to its high-pressure environment remain unknown. In this study, we investigated the elemental content of the exoskeleton of *H*. *gigas* specimens captured from the deepest points of the Mariana Trench. The *H*. *gigas* exoskeleton contained aluminum, as well as a major amount of calcium carbonate. Unlike other (accumulated) metals, aluminum was distributed on the surface of the exoskeleton. To investigate how *H*. *gigas* obtains aluminum, we conducted a metabolome analysis and found that gluconic acid/gluconolactone was capable of extracting metals from the sediment under the habitat conditions of *H*. *gigas*. The extracted aluminum ions are transformed into the gel state of aluminum hydroxide in alkaline seawater, and this gel covers the body to protect the amphipod. This aluminum gel is a good material for adaptation to such high-pressure environments.

## Introduction

The deepest bottom of the ocean is an extreme environment characterized by extra-high pressures, low temperatures, and oligotrophy, and few animals can adapt to such extreme environments [1–3]. The amphipod *Hirondellea gigas* is a resident of the deepest points of the Mariana Trench (Challenger Deep), the Philippine Trench, the Izu-Ogasawara Trench, and the Japan Trench, where it inhabits depths greater than 8,000 m [4–8]. We attempted to widely capture marine creatures using baited traps along deep-sea points, but these amphipods were the only catch [5, 6]. *H*. *gigas* produces a number of polysaccharide hydrolases as digestive enzymes and survives in oligotrophic environments by obtaining sugars from degradable plant debris by using such enzymes [6, 7]. However, the adaptation system to extra-high pressure is still unknown. The extra-high pressure in the deep sea affects the various chemical components of organisms. Calcium carbonate is an important component of crustacean exoskeletons; however, this component dissolves in seawater deeper than approximately 4,000–5,000 m (carbonate compensation depth, CCD) [9]; furthermore, crustaceans cannot migrate on the deep-sea floor below the CCD [10, 11]. Recently, a few species of crustaceans and foraminifera have been found in regions slightly deeper than the CCD [12–14]. Moreover, many foraminifera found at the bottom of the Challenger Deep are organic-walled allogromiids, which do not have a calcareous wall [15]. Because the habitable zone of *H*. *gigas* is at depths greater than 8,000 m, this species uses very little calcium carbonate in its exoskeleton. However, the mechanisms by which *H*. *gigas* adapts to its high-pressure environment remain unknown.

In this manuscript, we examined the chemical components of the exoskeleton of *H*. *gigas* specimens, which are affected by extra-high pressure. We identified aluminum and calcium carbonates in the exoskeleton though electron microscopy analysis. Aluminum is rarely present in seawater but present in large amounts in sediments. Thus, we examined the means by which *H*. *gigas* obtains aluminum from the sediment and found that gluconolactone/gluconic acid acts as the extractant of aluminum. Through a pressure experiment using *H*. *gigas* exoskeletons, we found that the aluminum protected calcium carbonate from the effects of high pressure.

## Materials and methods

### Amphipods

The deep-sea amphipod *Hirondellea gigas* was captured from the Challenger Deep in the Mariana Trench (11°22.11N, 142°25.86E, depth of 10,897 m) and the Izu-Ogasawara Trench (32°12.5766N, 142°08.0411E, depth: 9,450 m), as described in a previous manuscript [6, 7]. *H*. *gigas* is >3 cm from head to tail. We also purchased amphipods from Yokoebi-ya (Fukui, Japan). The coastal amphipods were captured from the seashore of Maizuru Bay (35°47.4331N, 135°39.5497E) in Japan, and their size was <2–3 mm from head to tail. All *H*. *gigas* and coastal amphipod specimens were stored in storage bags at -80°C without any selection. We randomly selected amphipods for all analyses.

### Sediment sample

We collected a sediment sample from the Challenger Deep in the Mariana Trench (11 22.030°N, 142 26.032°E, depth: 10,897 m) using the unmanned remotely operated underwater vehicle “KAIKO” on May 4, 1996. The sediment sample was stored at -80°C.

### Electron microscopy observations and energy-dispersive X-ray spectroscopy (EDS) analyses

#### Scanning electron microscopy (SEM) and EDS analysis

The freeze-dried amphipod sample was set on the stage of a scanning electron microscope, which was covered with a silicon plate and carbon tape to avoid energy-dispersive X-ray spectroscopy (EDS) signals from the stage. The exoskeleton of the amphipod was observed with scanning electron microscopy (SEM) (SU6600, Hitachi High-Technologies Co., Tokyo, Japan) under an accelerating voltage of 20 kV, and the elementary components were analyzed by EDS (X-Max^N^, Oxford).

#### Scanning transmission electron microscopy (STEM) and EDS analysis

We removed exoskeletons from *H*. *gigas* and washed them with deionized distilled water (DDW) and ethanol to avoid interference from oil components during electron microscope observations. Pieces of *H*. *gigas* exoskeleton obtained after milling were placed on a transmission electron microscopy (TEM) grid (200 mesh Cu Formvar/carbon-coated grid, JEOL) and observed by TEM (JEM-2100, JEOL) with an accelerating voltage of 200 kV. Scanning TEM (STEM)-EDS analysis was performed at 200 kV with an accumulation time of 60 s.

### Identification of calcite using X-ray powder diffraction (XRD) analysis

The exoskeletons of *H*. *gigas* were removed from individuals with tweezers and dissecting scissors and washed with methanol and chloroform. After drying the exoskeletons, we cut and crushed them into powder for X-ray powder diffraction (XRD) analysis. The exoskeleton powder was analyzed by an X-ray diffractometer (SmartLab, Rigaku) with a Cu radiation source (Kα = 1.5418 Å) at 45 kV and 200 mA. The 2θ scan speed, step width, and range were 21.6746 deg/min, 0.02 deg, and 20 to 50 deg, respectively. Calcite was identified in the exoskeleton powder through a database (International Center for Diffraction Data (ICDD)) search of the obtained peak positions.

### Preparation of metal and gluconolactone/gluconic acid solution from amphipods

To measure the content of metal ions and gluconolactone/gluconic acid, each component was extracted from the amphipods. We carefully removed the exoskeletons from the amphipods with tweezers and dissecting scissors. Then, we subdivided the exoskeleton in 0.1 N sodium acetate buffer (pH 4.0) and stirred this suspension with a vortex mixer to extract the metal ions and gluconolactone/gluconic acid from the exoskeleton. After centrifugation (2,000 x g, 10 min, 4°C), the supernatant was collected, and the remaining parts of exoskeleton were suspended in the same buffer. Then, we repeated the stirring and centrifugation of the suspension. Both supernatants were collected and used for the measurement of metal ions and gluconolactone/gluconic acid in the exoskeleton. The remaining body was also subdivided in 0.1 N sodium acetate buffer (pH 4.0) and mashed by a BioMasher II (Nippi Inc, Tokyo, Japan) to extract metal ions and gluconic acid/gluconolactone. A small amount of the solution that had leaked from the amphipod in the process of removing the exoskeleton was also added. After centrifugation (5,000 x g, 10 min, 4°C), the supernatant was divided into two layers of oil and water. Each layer was collected separately. Then, the precipitate was suspended in the same buffer and mashed again. After centrifugation (5,000 x g, 10 min, 4°C), each oil and water layer was collected separately. We combined each of these layers with the corresponding layers obtained from the first extraction and used the results for measurements.

### Measurement of aluminum, iron, and gluconic acid/gluconolactone content

The aluminum contents of the amphipod extract and water were measured using fluorometric analysis with 8-quinolinol [16]. We suspended samples in DDW, added 0.2 ml of 1% (wt./vol.) 8-quinolinol (Nacalai Tesque, Kyoto, Japan) and 0.2 ml of 2 N CH_3_COONa, and then added DDW to a volume of 5 ml. After mixing well, the aluminum 8-quinolinol complex was extracted with 1 ml of chloroform. The aluminum content was measured by the fluorescent intensity (excitation: 360 nm, emission: 535 nm). An aluminum chloride solution (Wako Pure Chemical Industries, Ltd.) was used as the reference. The iron content of the extracted sediment or soil was measured at an absorbance of 510 nm using a Pack Test Fe (Kyouritu Chemical-Check Lab. Co., Tokyo, Japan) based on the reaction of the Fe^2+^ ion and *o*-phenanthroline after reduction. An iron(II) chloride solution was used as a reference. The D-gluconic acid/gluconolactone content of the amphipods was measured using a “gluconic acid/D-glucono-∂-lactone” E-kit (R-Biopharm AG, Damstadt, Germany). Sodium gluconic acid (Wako Pure Chemical Industries, Ltd.) was used as a reference. We calculated the total amount of aluminum or gluconic acid/gluconolactone in each amphipod from the measured contents and volumes of the amphipod extracts.

### Degradation of gluconolactone/gluconic acid in *H*. *gigas* extract

We crushed and mashed an *H*. *gigas* individual with a BioMasher II (Nippi Inc., Tokyo, Japan). The mashed sample was centrifuged (3,000 x g, 10 min 4°C), and a body fluid sample was collected. The precipitate was washed with 0.2 ml of DDW, and the supernatant was collected and combined with the body fluid sample after centrifugation (3,000 x g, 10 min 4°C). The combined sample was filtered to remove protein using an Amicon Ultra 3K device (Merck, Darmstadt, Germany). The pH and volume of the filtered sample were adjusted to 8.0 with 0.1 N NaOH and 0.5 ml, respectively. A sample volume of 0.1 ml and the same volume of enzyme reaction solution consisting of 0.2 M Tris-HCl buffer containing 20 mM ATP and 5.0 mM NADP were mixed, and then, 1 U of gluconate kinase and 10 U of 6-phosphogluconate hydrogenase (R-Biopharm AG, Darmstadt, Germany) were added. The enzyme reaction was carried out at 25°C for 20 min. The reaction was stopped by filtration with a 3K Amicon Ultra 0.5 ml filter. The pH of the filtrate was adjusted to approximately 5.0 by the addition of HCl. A control reaction was also carried out without enzymes.

### Extraction of aluminum from the sediment of the Mariana Trench under high pressure

A sample volume of 0.1 ml was mixed with sediment from the Mariana Trench (0.1 g dry weight) in 0.1 M sodium acetate buffer (pH 5.0). We incubated the mixture for 2 h at 4°C and 100 MPa. After centrifugation of the mixture (5,000 x g, 10 min, 4°C), the aluminum content of the supernatant was measured.

### Measurement of Ca ion release from the exoskeleton of *H*. *gigas* after a high-pressure experiment

We cut out the exoskeleton from a *H*. *gigas* body and divided the exoskeleton into two pieces. One piece was washed twice with ice-cold DDW to remove adhered Al gel. The other piece was washed twice with ice-cold artificial seawater (NaCl, 20.7 g; MgCl_2_6H_2_O, 9.4 g; CaCl_2_2H_2_O, 1.3 g; Na_2_SO_4_, 3.5 g; KCl, 0.6 g; NaHCO_3_, 0.17 g; KBr, 85 mg; Na_2_B_4_O_7_10H_2_O, 34 mg; SrCl_2_, 12 mg; NaF, 3 mg; LiCl, 1 mg; other trace elements, <0.1 mg per liter) (Nihon Pharmaceutical Co., Ltd, Tokyo, Japan). A piece of exoskeleton was placed into a 1.6 ml plastic tube filled with ice-cold artificial seawater. After sealing with Parafilm M (Bemis Flexible Packaging, Neenah, WI, USA), we pressurized the packed samples to 100 MPa for 24 h at 2°C. Then, we measured the Ca ion concentration dissolved in the artificial seawater with Ca ion assay kit (OCPC) (Metallogenics Co., Ltd, Chiba, Japan) [17].

### Animal research

We obtained permission from the government of the Federated States of Micronesia to capture animals from the Mariana Trench (PERMIT NO. FM09-RV00083RS-01). Because the Izu-Ogasawara Trench is located in the exclusive economic zone (EEZ) of Japan, permission to catch animals at this Trench was not necessary.

*Hirondellea gigas* is not listed in the IUCN Red List of Threatened Species. We confirmed that *H*. *gigas* is not an endangered or protected species.

## Results

### Scanning electron microscopy (SEM)/energy dispersive X-ray spectrometry (EDS) analysis of *H*. *gigas* exoskeleton

**Fig 1:**
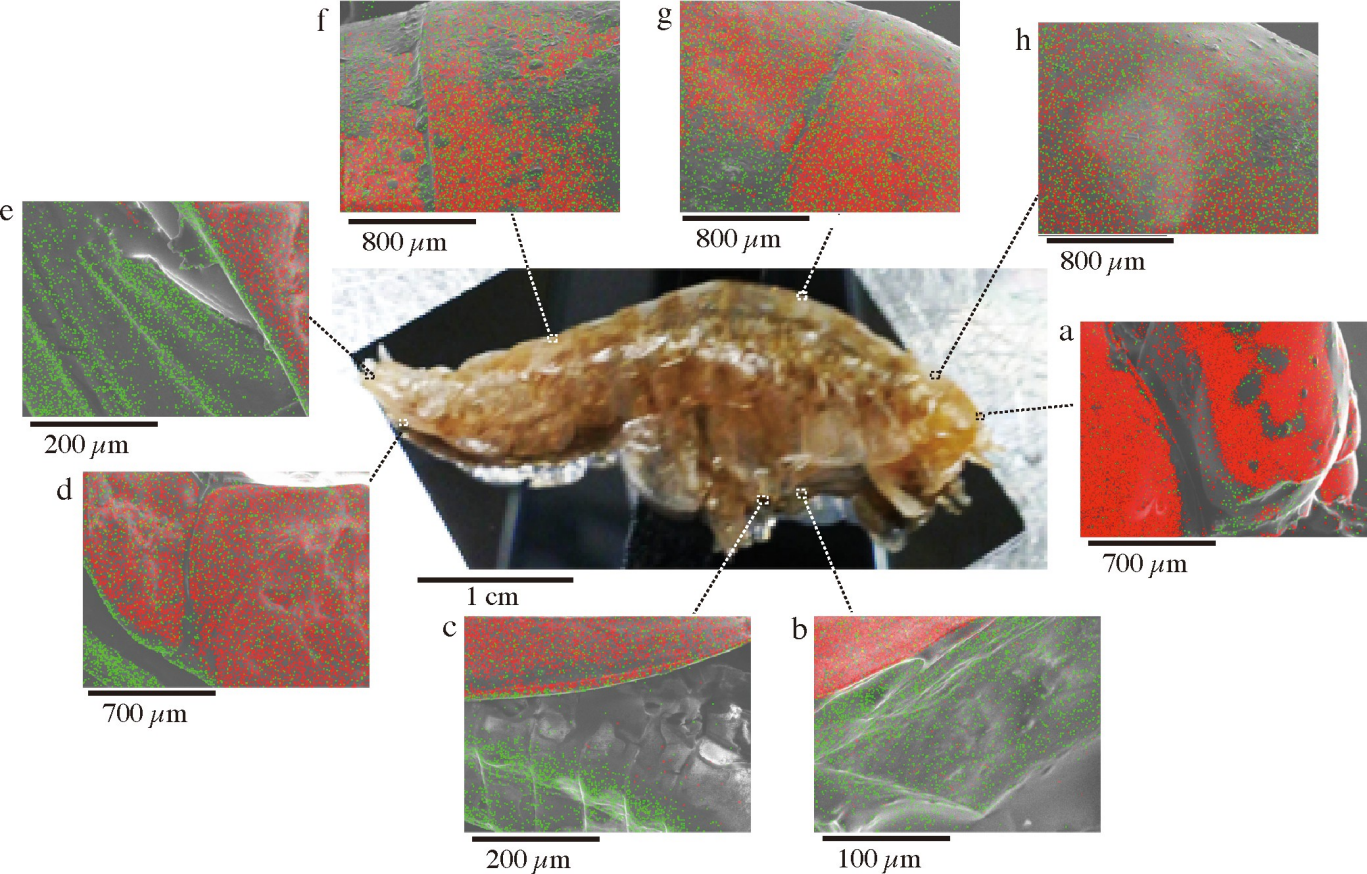
SEM/EDS analysis of the exoskeleton of *H*. *gigas*. *H*. *gigas* specimens captured from the Challenger Deep were freeze-dried for SEM observations. The SEM observations were conducted without any coating. Calcium (red) and aluminum (green) are mapped on the SEM images (A, a-h). The EDS spectrum includes an annotation of each element with its Kα energy levels (C: 0.284, O: 0.532, Na: 1.071, Mg: 1.253, Al: 1.486, P: 2.013, S: 2.307, Cl: 2.621, Ca: 3.69 (keV)).

**Fig 2:**
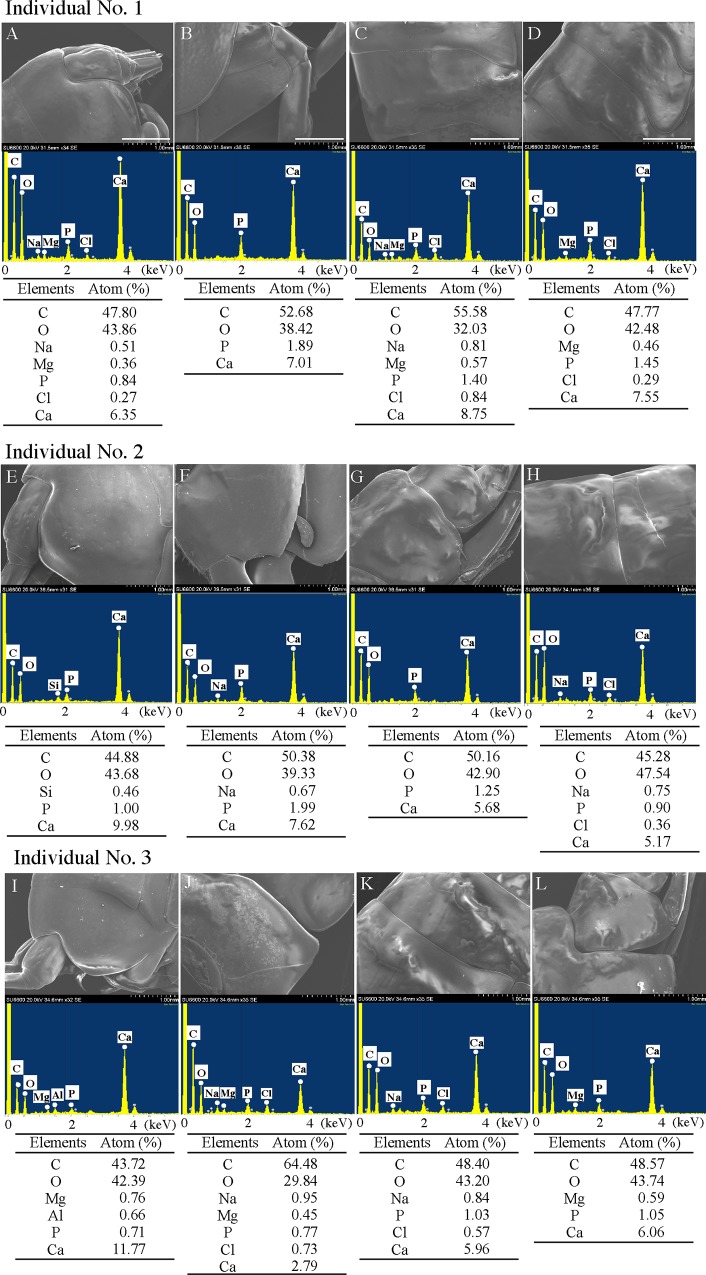
SEM/EDS analysis of the exoskeleton of *H*. *gigas* after washing with DDW. Three *H*. *gigas* specimens captured from the Challenger Deep were washed 3 times with DDW and freeze-dried for SEM observations. The SEM observations were conducted without any coating. Exoskeleton parts of the head (A, E, I), the body (B, F, J), the back (C, G, K), and the telson (D, H, L) were observed. Each panel shows an SEM image (top), EDS spectrum (middle), and element composition obtained from the EDS spectrum (bottom).

First, we analyzed the elements of the exoskeletons of *H*. *gigas* captured from the Challenger Deep in the Mariana Trench using SEM/EDS. Our results showed a peak of aluminum, as well as peaks of other major metals, such as calcium, magnesium, potassium, and sodium, in various parts of the exoskeleton (Fig 1 and Table 1). Calcium was the most abundant metal ion. Aluminum was widely distributed throughout the entire body, and an especially high content of aluminum was observed in the tail (telson) and along the edge of the feet (pereopods, or uropods) (Fig 1 and S1 Fig). However, silicon, which is the major component of the clay mineral aluminosilicate [18, 19], was not observed; therefore, the aluminum in the exoskeleton was not derived from aluminosilicate in sediment. We repeated the same analysis with other *H*. *gigas* individuals and obtained similar results, including a high aluminum content in their telsons (S1 and S2 Figs). We analyzed a total of 6 individuals from all captured 185 individuals using SEM/EDS and found aluminum and calcium in the exoskeleton. Similar to the amphipods from the Mariana Trench, the *H*. *gigas* specimens captured from the Izu-Ogasawara Trench also had aluminum in their exoskeleton (S3 Fig). A total of 3 individuals randomly selected from approximately 400 individuals from the Izu-Ogasawara Trench were analyzed using SEM/EDS, which revealed aluminum and calcium in the exoskeleton. To identify whether the aluminum was accumulated in the exoskeleton or adhered to the surface of the exoskeleton, we observed the presence or absence of aluminum after washing the surface of *H*. *gigas* individuals with DDW. The aluminum was clearly removed from the exoskeleton (Fig 2). Therefore, the aluminum covered the surface of the exoskeleton rather than accumulating in the internal part of exoskeleton. A comparison was performed to investigate the aluminum in the exoskeleton of a shallow-sea coastal amphipod that was captured from Maizuru Bay in Japan and identified as *Prontogenesia* sp. based on the amino acid sequence of cytochrome oxidase (S4 Fig). In contrast to the results for the deep-sea amphipods, the SEM/EDS analysis of the exoskeletons of *Prontogenesia* sp. did not show an aluminum peak (S5 Fig). The coastal amphipod *Prontogenesia* sp. did not have aluminum in its exoskeleton. To date, aluminum content has not been reported in the exoskeletons of crustaceans; however, we contend that an aluminum-containing exoskeleton is a unique property of hadal amphipods.

**Table 1:** Ratio of atoms calculated from the total spectrum count* in each panel in Fig 1.

Atomic (%)	Panel in Fig 1							
	a	b	c	d	e	f	g	h
C	69.43	53.82	67.33	55.32	63.91	67.06	57.20	55.46
N	0.00	0.00	0.00	0.00	0.00	0.00	2.29	0.00
O	25.11	34.78	21.65	34.95	24.26	28.85	35.41	38.25
Na	0.40	0.58	0.53	0.57	0.50	0.66	0.81	0.82
Mg	0.17	0.58	0.34	0.73	0.44	0.18	0.33	0.36
Al	0.07	3.33	1.27	0.53	3.10	0.03	0.03	0.05
P	0.39	0.64	0.82	0.19	0.13	0.32	0.38	0.51
S	0.00	0.00	0.00	1.60	0.00	0.11	0.16	0.12
Cl	0.33	0.41	0.72	0.57	0.61	0.33	0.40	0.41
K	0.06	0.00	0.07	0.10	0.00	0.02	0.03	0.04
Ca	4.04	5.86	7.25	5.45	6.11	2.44	2.96	3.99

*) The total spectrum counts were as follows: panel a: 321,710; panel b: 235,772; panel c: 235,233; panel d: 319,672; panel e: 171,886; panel f: 585,772; panel g: 540,040; and panel h: 400,863.

### STEM/EDS analysis of *H*. *gigas* exoskeleton

**Fig 3:**
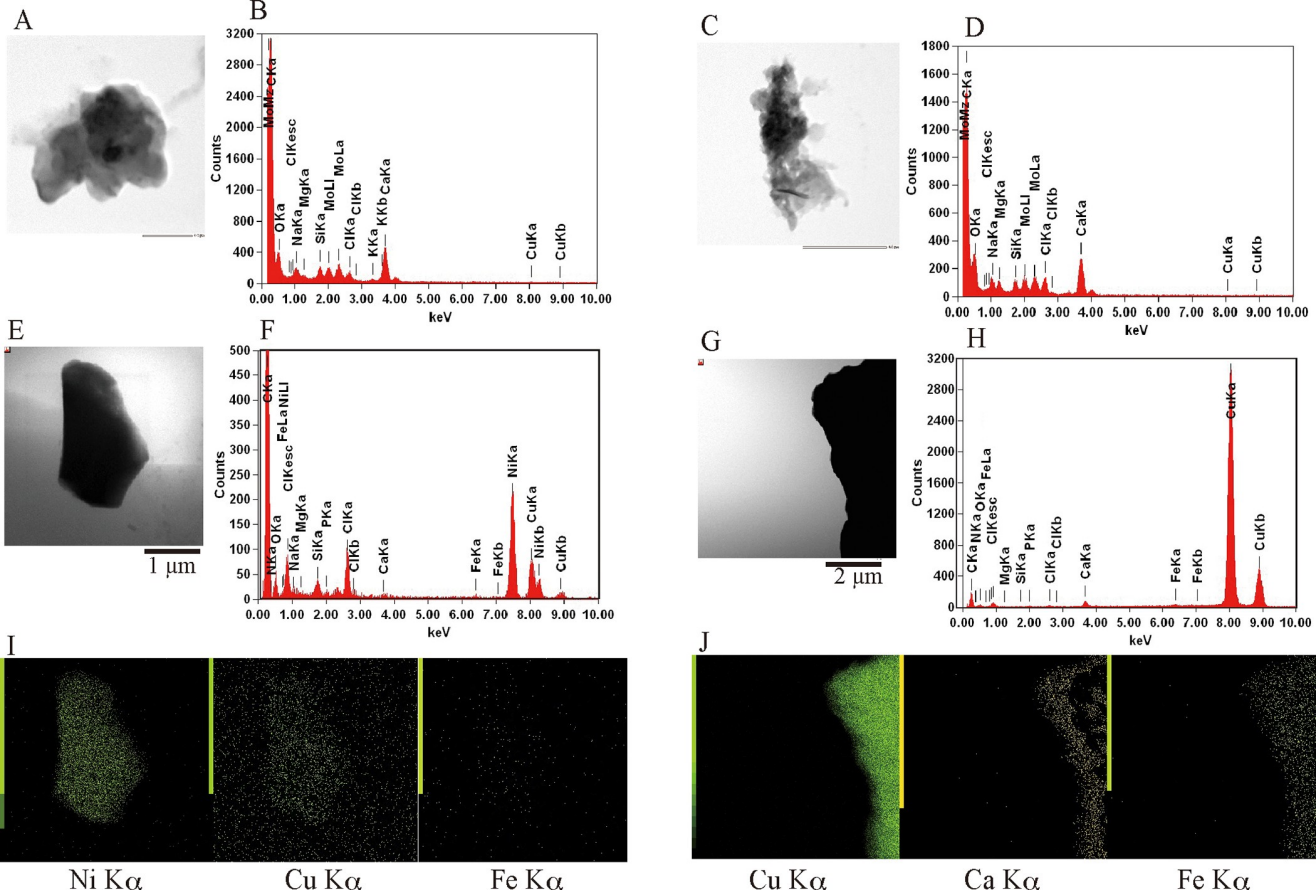
STEM/EDS analysis of pieces of *H*. *gigas* exoskeleton. Exoskeletons of *H*. *gigas* captured from the Challenger Deep were removed from the individuals, freeze dried, and then scraped. Bright-field STEM observations were conducted for pieces of the exoskeletons (A, C, E, G). Characteristic X-ray patterns were collected over 60 s (B, D, F, H), and the major metal signals from panels E and G were mapped (I, J). The observed Cu and Mo signals were caused by the TEM grid. The Si signal was background.

We also observed crushed exoskeletons through STEM/EDS to find aluminum in the internal exoskeletons of *H*. *gigas* captured from the Challenger Deep (Fig 3). We found calcium in all crushed exoskeleton, and nickel or copper in a few pieces of exoskeleton. Copper and nickel are minor elements in marine sediment, and *H*. *gigas* does not accumulate sufficient amounts for detection in SEM/EDS analysis. Aluminum was not contained in the internal exoskeleton. Silicate and molybdenum were observed as background signals from sample holder. We also observed the internal exoskeletons of amphipods captured from the Izu-Ogasawara Trench and did not found any aluminum peak in STEM/EDS analysis (S6 Fig).

### XRD analysis of *H*. *gigas* exoskeleton

**Fig 4:**
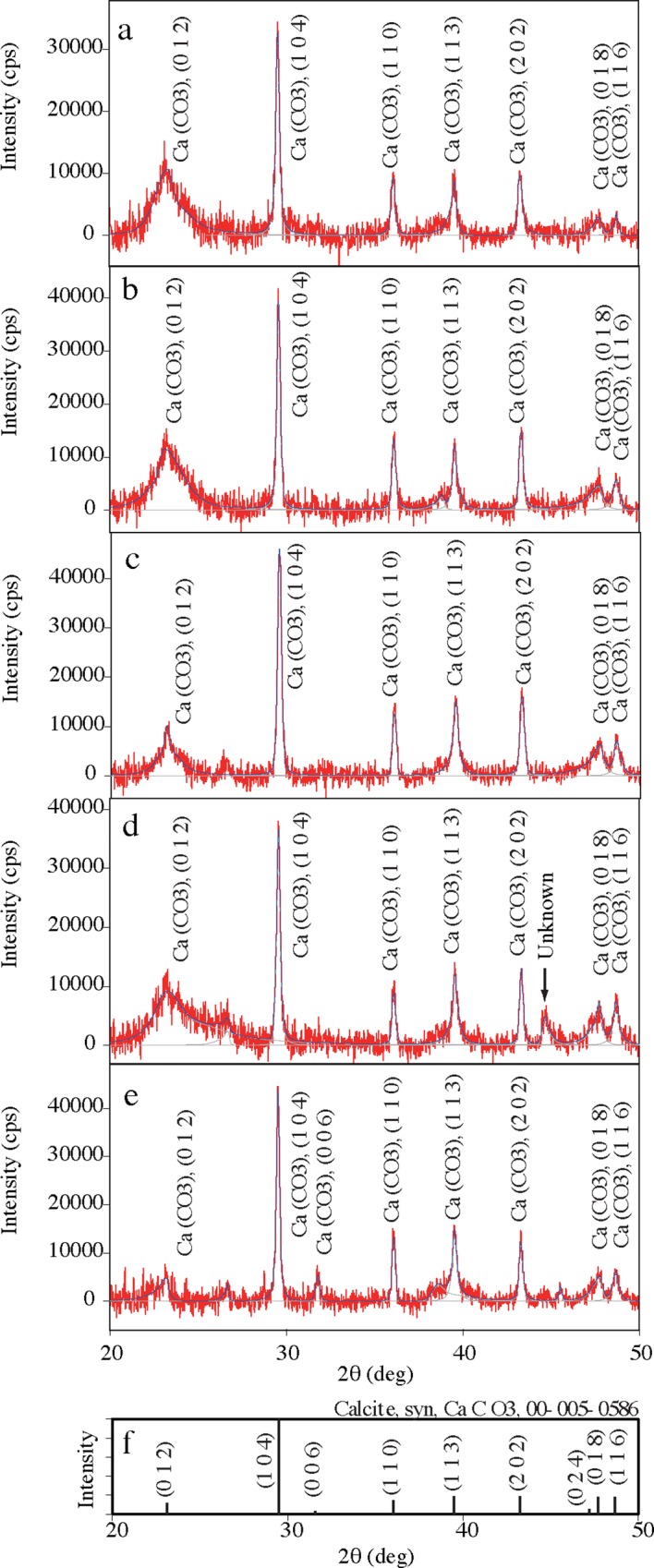
XRD analysis of *H*. *gigas* exoskeleton. Exoskeleton samples prepared from 5 individuals of *H*. *gigas* were used for XRD analysis as described in the Materials and Methods (panel A-E). The annotations of peaks were obtained from a database search (panel F). The arrow in panel d indicates an unknown peak, which was suggested to be AlO(OH) from a library search. We could not identify the peak as AlO(OH) because other minor peaks were not found.

Through EDS analysis, we found that calcium was the major metal in the exoskeleton of *H*. *gigas*. Generally, calcium occurs as calcium carbonate and calcium phosphate in crustaceans [20–24]; however, a much lower peak of phosphorus than that of calcium was detected in the exoskeletons of *H*. *gigas* whose habitat is much deeper than the CCD (Table 1). This result indicates the possibility of the existence of calcium carbonate in the exoskeleton. Thus, we carried out XRD analysis of the exoskeletons of 5 randomly selected individuals to examine the chemical and physical state of calcium (Fig 4). The diffraction peak positions in the 5 samples were the same except for one unknown peak. The 2θ positions of the 7 main peaks were 23.1±0.1, 29.5, 36.0, 39.5, 43.2, 47.7, and 48.6. The observed peaks were indexed to calcite according to the standard ICDD card No. 00-005-0586. Then, the crystal material in the exoskeleton was suggested to be trigonal calcium carbonate (calcite). In contrast, one unknown peak (2θ = 44.6) was found in one sample and suggested to be AlO(OH) (1 1 1) (panel d). Since we could not find the largest peak of AlO(OH) (1 1 0) (2θ = 22.3), we did not conclude that this peak originated from AlO(OH).

### Aluminum content in *H*. *gigas*

Next, we measured the content of aluminum in *H*. *gigas* individuals. To avoid the influence of aluminum oxide originating from sediment, we used the 8-quinolinol method and applied an 8-quinolinol-aluminum ion complex as a fluorescent label [16]. We selected three individuals captured from the Challenger Deep in the Mariana Trench (S7 Fig). The exoskeletons of the three *H*. *gigas* individuals contained over 50% aluminum and exhibited only minor differences in content (S1 Table), whereas the body fluid presented varying aluminum contents among the 3 individuals. Because crustaceans inhabiting contaminated areas accumulate metals in their gut [25, 26], the presence of aluminum in the exoskeleton was not a result of simple accumulation.

### Identification of the aluminum extraction agent in *H*. *gigas*

**Fig 5:**
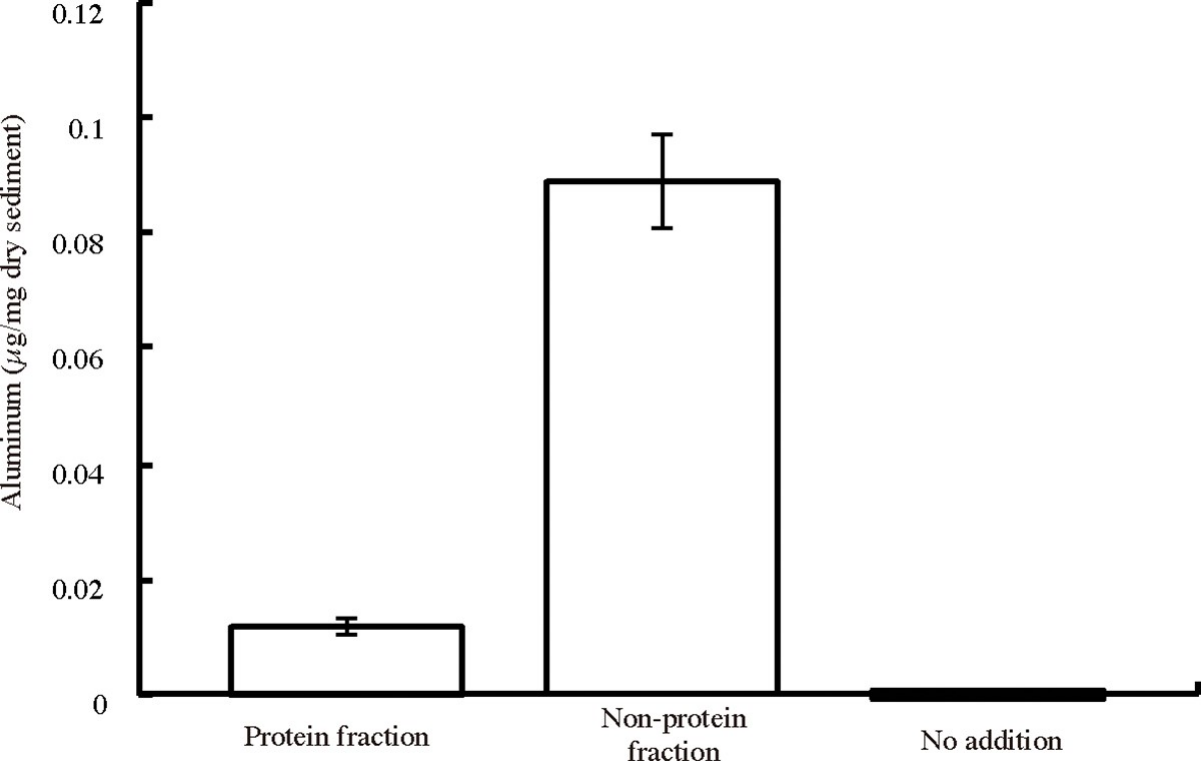
Extraction of aluminum from the sediment of Challenger Deep by *H*. *gigas* protein and nonprotein fractions. Three *H*. *gigas* individuals were scrapped and then suspended in 1 ml of (NH_4_)_2_SO_4_ solution at 80% saturation. After incubation at 4°C for 2 h, the *H*. *gigas* suspensions were centrifuged at 20,000 x *g* for 30 min. The supernatants were used as nonprotein fractions, and the precipitates were suspended in 1 ml of DDW to prepare protein fractions. We mixed 300 μl of each fraction with the sediment suspension and added sodium acetate buffer to a final volume of 1.8 ml (final concentration: 50 mM, pH 5.0). The mixtures were then pressurized at 100 MPa and incubated at 2°C. After 1 h incubation, the mixtures were depressurized and centrifuged at 20,000 x *g* for 10 min. The aluminum content of the supernatants was measured as described in the Materials and Methods section. The error bar shows the S.D. (n = 3).

**Fig 6:**
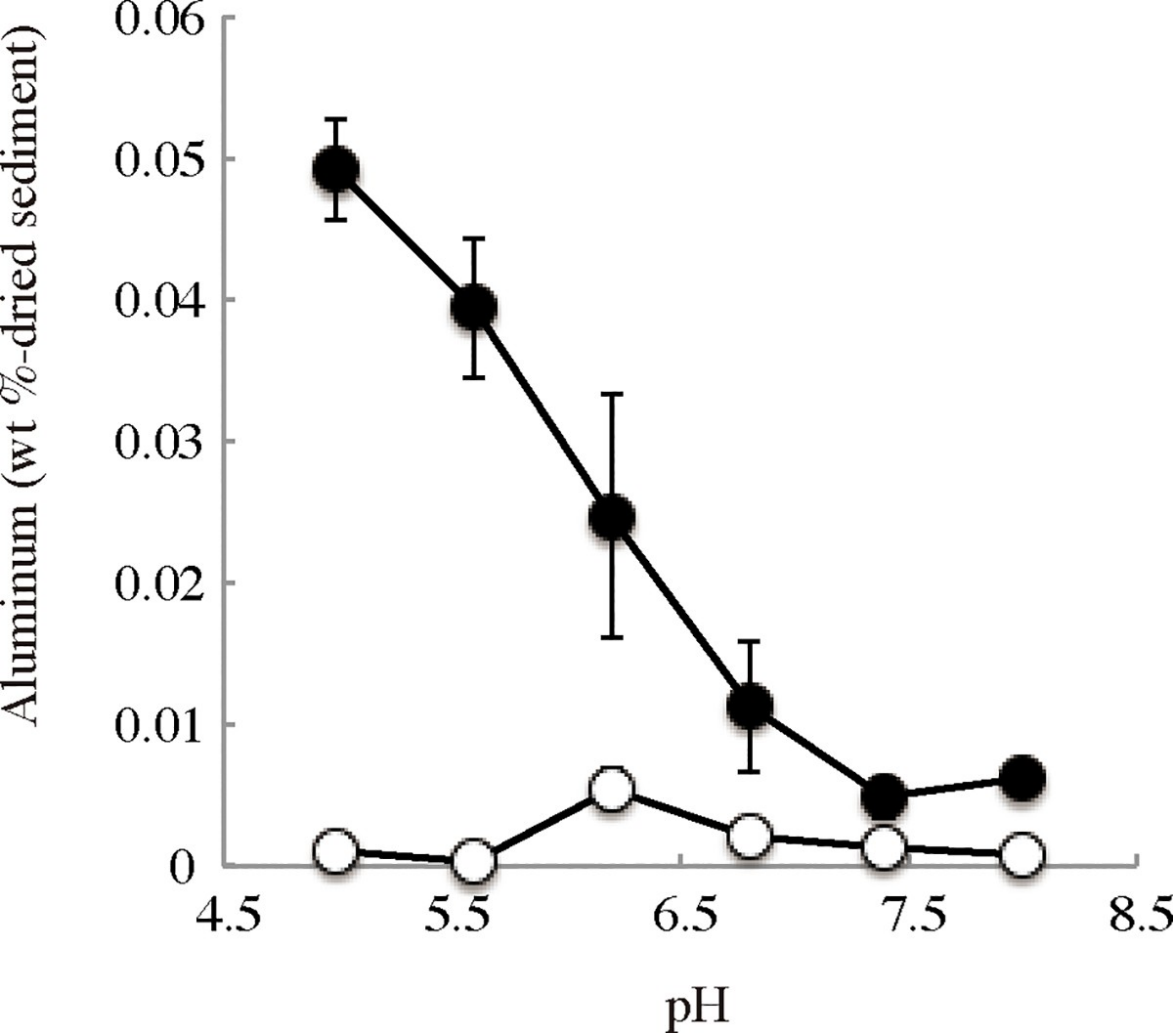
Extraction of aluminum from the sediment of the Challenger Deep by gluconic acid/gluconolactone. Sediment samples were washed with DDW five times and then suspended in buffers containing 10 mM sodium gluconic acid/gluconolactone (closed circle) or none (open circle). The extracted aluminum was measured as described in the Materials and Methods.

Because aluminum is the third-most abundant element on Earth, possible origins of the aluminum in *H*. *gigas* include marine sediment and seawater. A large amount of aluminum occurs as aluminum oxide in clay minerals in marine sediment [18, 19, 27]. However, aluminum ions have been found in deep-sea water in small amounts (approximately 2 nM) in the North Pacific [28, 29]. When deep-sea amphipods were caught in baited traps, the digestive organs contained used bait, sediment, or nothing [5, 6]. We expected that *H*. *gigas* extracts aluminum from the sediment. SEM/EDS analysis of another *H*. *gigas* individual showed the presence of aluminum and silica in the head part, and element mapping showed the same distribution of aluminum and silicon in the head (S8 Fig). The atomic percents calculated from the peaks were C: 49.2, N: 14.1, O: 30.5, Na: 0.42, Mg: 0.25, Al: 0.47, Si: 1.66, P: 0.22, Cl: 0.32, and Ca: 5.1. The ratio of aluminum to silicon was approximately 1:3.53, which was similar to the aluminum ratio in the sediment (1:3.34–1:4.96) (S2 Table) [30]. Accordingly, the aluminum found in *H*. *gigas* is likely dependent on the release of aluminum from marine sediment under the acidic conditions of the gut, whose pH was estimated approximately 5–6 from the digestive enzyme activities [6, 7, 31]. However, only a limited amount of aluminum was released from the sediment of the Challenger Deep under the same physical conditions as observed in the *H*. *gigas* gut or habitat (2°C, 100 MPa, and pH 5.0–8.0) [6, 7]. To examine the extraction agent of aluminum from the sediment, we performed aluminum extraction using the protein and nonprotein fractions of crushed *H*. *gigas*, which correspond to enzymatic and chemical reactions related to aluminum extraction, respectively. The nonprotein fraction could extract aluminum (Fig 5); therefore, we conducted a metabolome analysis of an entire *H*. *gigas* individual to identify the chemicals that contribute to extracting aluminum from the sediment (see SI text). The results indicated that *H*. *gigas* produces 217 chemicals (S3 Table). To determine potential candidates, we focused on 60 chemicals that are not involved in the metabolic pathways of the animal because metabolites involved in these pathways usually occur in the inner cells and cannot react to extracellular marine sediment in the gut. Among the 60 chemicals, gluconic acid/gluconolactone (gluconolactone dominates in acidic pH) is a strong organic chelating agent [32]. A high glucose content (0.43%±0.1% (w/w) (dry weight)) was present in the *H*. *gigas* individuals; glucose is a source of gluconic acid/gluconolactone [6]. Furthermore, gluconic acid/gluconolactone levels of 0.3–0.4 mM were measured in the bodies of the *H*. *gigas* presented here (S4 Table). We examined the effects of gluconic acid/gluconolactone on the release of aluminum from the sediment of the Challenger Deep and confirmed that gluconic acid/gluconolactone released aluminum ion from the sediment at pH 6.2 or lower under *in situ* conditions (Fig 6). Under acidic conditions, gluconolactone is the main component in the chemical equation of gluconic acid/gluconolactone and acts as an extraction agent of aluminum from sediment in the gut [33]. Therefore, the extraction of aluminum is a unique property of gluconolactone, rather than a chelating activity of gluconic acid. When we removed gluconic acid/gluconolactone from *H*. *gigas* extract by the enzymatic reaction of gluconokinase (EC 2.7.1.12), the ability to extract aluminum from sediments was lost (S9 Fig). Gluconolactone is the main aluminum extractor in *H*. *gigas*. Gluconic acid/gluconolactone can extract minor amounts of iron from sediment under the same deep-sea bottom conditions (S10 Fig). Therefore, copper and nickel, found in STEM/EDS observation, were also included in and extracted from the sediment by gluconic acid/gluconolactone [19, 32].

### Effect of high pressure on the release of calcium ion from the exoskeleton

**Fig 7:**
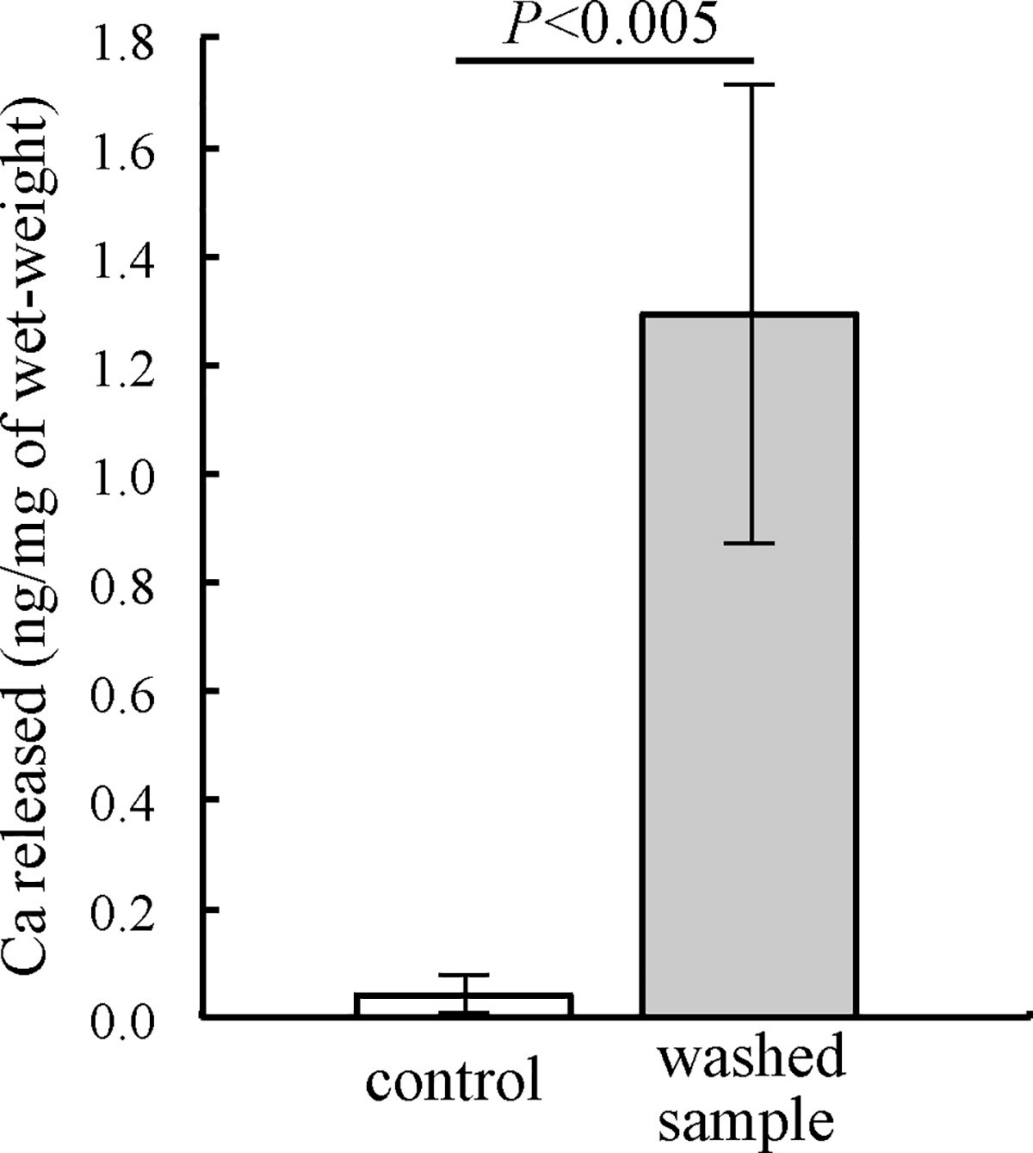
The release of calcium ion from the exoskeleton of *H*. *gigas* under 100 MPa. The exoskeleton was removed from 5 individuals. After dividing the exoskeleton into two pieces, the wet weight of each piece was measured. One piece was washed with DDW (washed sample), and the other piece was not washed (control). Both samples were pressurized under 100 MPa in artificial seawater at 2°C for 24 h. The released calcium was measured as described in the Materials and Methods. The error bars show the SD (n = 5). The results of the t-test confirmed that the impairment was significant (*p*<0.005).

To investigate the role of the aluminum hydroxide gel in a high-pressure environment, we examined the release of calcium ion from the exoskeleton in the presence or absence of aluminum gel under 100 MPa in artificial seawater (Fig 7). The exoskeleton of *H*. *gigas* was prepared as described in the Materials and Methods. The amount of calcium ion released from the non-Al gel exoskeleton was up to 1.29 ± 0.42 (SD., n = 5) (ng/mg of wet-weight exoskeleton). In contrast, a very small amount of calcium ion was released from the native exoskeleton (0.042 ± 0.036 (SD., n = 5) (ng/mg wet weight exoskeleton)). No calcium ion was released from either exoskeleton sample under 0.1 MPa. The Al gel protected the elution of calcium ion from the exoskeleton under high pressure.

## Discussion

In this study, we have shown that aluminum hydroxide gel covers the body of *H*. *gigas*. *H*. *gigas* inhabits the bottom of the deepest trench by obtaining glucose, a resource of gluconolactone/gluconic acid, from plant debris buried in sediment and digesting the glucose with its own cellulase and hemicellulose hydrolases [5–7]. Namely, *H*. *gigas* has ability to take in both aluminum resources and aluminum extraction agents from the sediment. Aluminum hydroxide gel is constructed by the chemical behavior of aluminum ion in response to pH. The extracted aluminum ions in the gut are released to the seawater at approximately pH 8, where the ions are transformed to the gel state of aluminum hydroxide and then mainly stored in the *H*. *gigas* telson [34, 35]. The aluminum is spread on the surface of the exoskeleton without being discharged with excrement. Thus, it is thought that *H*. *gigas* has some aluminum transport system. We also detected gluconic acid in the exoskeletons (S4 Table); gluconolactone turns into gluconic acid when in contact with alkaline seawater. Hence, there should be a transporter of aluminum in *H*. *gigas*. The converted gluconic acid would work as a chelating agent to transfer aluminum ions to the whole exoskeleton, although the binding properties and stability of aluminum hydroxide gel with respect to organisms are still unclear. The aluminum hydroxide gel is stable only in an alkaline environment and would be present only in specimens inhabiting the ocean and some alkaline lakes.

Fig 7 clearly shows that the aluminum gel protected calcite in the exoskeleton; however, removing the aluminum gel and/or washing the exoskeleton with DDW may cause leakage of wax esters in the exoskeleton. It has been reported that the organic components in the exoskeleton are important to stabilize calcite in the exoskeleton [36]. The leaking of calcium from the DDW-washed exoskeleton would depend on the removal of aluminum gel as well as the organic components in the exoskeleton. Aluminum gel and organic components would act as a shield against high pressure and keep calcite in the exoskeleton to enable *H*. *gigas* to survive on the deepest sea floor.

We also carried out a preliminary experiment to determine the protective effect of aluminum hydroxide gel under high-pressure stress using salmon roe and found less protein leakage and no color change in the presence of aluminum hydroxide gel, even under 100 MPa (S11 Fig). In addition to providing calcite protection, the aluminum gel may contribute to relieving stress caused by high pressure. The mechanism by which *H*. *gigas* has adapted to deep-sea environments is done through the extraction and utilization of aluminum, which is summarized in S12 Fig.

## Supporting information

S1 Fig SEM/EDS analysis of the exoskeleton of *H*. *gigas* telson. *H*. *gigas* specimens captured from Challenger Deep was freeze dried for SEM observations (A, B). Panel B shows an enlargement of the red square in panel A. SEM observations and EDS analyses were conducted without any coating. The EDS spectrum of panel B includes an annotation of each element with its Kα energy level (C: 0.284, O: 0.532, Na: 1.071, Mg: 1.253, Al: 1.486, P: 2.013, S: 2.307, Cl: 2.621, Ca: 3.69 (k eV)) (C). The total spectrum counts were 117,388 in the EDS analysis, and the major elements were mapped (D).
(PDF)

S2 Fig SEM/EDS analysis of the *H*. *gigas* exoskeleton. An SEM/EDS analysis was conducted on the telson region (A) and the exoskeleton (E) as described in the Methods section. The EDS spectra of panel A and E include annotations of each element with its Kα energy level (C: 0.284, O: 0.532, Na: 1.071, Mg: 1.253, Al: 1.486, P: 2.013, S: 2.307, Cl: 2.621, Ca: 3.69 (k eV)) (B, F). The metal peaks were mapped for the telson (C) and the exoskeleton (G). The composition of elements was calculated from the total spectrum counts (D: 320,357, H: 141,002).
(PDF)

S3 Fig SEM/EDS analysis of *H*. *gigas* captured from the Izu-Ogasawara Trench. An SEM/EDS analysis was conducted on *H*. *gigas* captured from the Izu-Ogasawara Trench. Four views of the exoskeleton were analyzed (A, E, I, M) as described in the Methods section. Panels E, I, and M were observed with accelerating voltages of 15 kV, because oil components induce sample charging and cause drift in SEM/EDX images, which cannot be suppressed at high acceleration voltage sufficiently. Only calcium and aluminum were mapped (B, F, J, N). The EDS spectrum includes an annotation of each element with its Kα energy level (C: 0.284, O: 0.532, Na: 1.071, Mg: 1.253, Al: 1.486, P: 2.013, S: 2.307, Cl: 2.621, Ca: 3.69 (k eV)). All EDS signals were detected and calculated from the total spectrum counts (C and D, G and H, K and L, O and P).
(PDF)

S4 Fig Phylogenetic tree of the captured coastal amphipods and related amphipods reconstructed from the mitochondrial COI protein sequence alignment (192 amino acids). The COI genes were amplified and cloned in *E*. *coli* DH5α as described in the Methods section. Then, we decided DNA sequences of 6 *E*. *coli* clones. The amino acid sequence of the COI obtained from the coastal amphipods indicated “clone1-6” in this study. The amino acid sequences of the COI of related amphipods were obtained from GenBank, and each accession number was added after the species name. Bold lines indicate bootstrap support above 95% as inferred from the maximum likelihood analysis. The scale bar for the branch length is denoted by the estimated number of amino acid substitutions per site.
(PDF)

S5 Fig SEM/EDS analysis of the coastal amphipods. Three amphipods were freeze dried and then analyzed. The telson (A, C) and foot (E) of *H*. *gigas* contained aluminum. Panel A and B were observed with accelerating voltages of 10 kV because of sample movement related to the oil component. EDS analysis was conducted as described in the Methods section (B, D, F). The EDS spectrum includes an annotation of each element with its Kα energy level (C: 0.284, O: 0.532, Na: 1.071, Mg: 1.253, P: 2.013, S: 2.307, Cl: 2.621, Ca: 3.69 (k eV)). The peak of Si was obtained from the backfield in F.
(PDF)

S6 Fig STEM/EDS analysis of pieces of the deep-sea amphipod’s exoskeleton. Exoskeletons of the amphipods captured from the Izu-Ogasawara Trench were removed from the individuals, freeze dried, and then scrapped. Bright-field STEM observations were conducted for pieces of the exoskeletons (A, C, E). Characteristic X-rays were collected over 60 s (B, D, F). The Cu or Mo signals were caused by the TEM grid. The Si signal was background.
(PDF)

S7 Fig *H. gigas* individuals used for the aluminum measurements. Deep-sea amphipod *H*. *gigas* individuals were immediately frozen and maintained at -80°C after capture from Challenger Deep. These amphipods were selected randomly from frozen stock.
(PDF)

S8 Fig SEM/EDS analysis of the exoskeleton of the head of *H*. *gigas*. *H*. *gigas* specimens captured from Challenger Deep were freeze dried for the SEM observations (A). SEM observations were conducted without any coating. The EDS spectrum included an annotation of each element with its Kα energy level (C: 0.284, O: 0.532, Na: 1.071, Mg: 1.253, Al: 1.486, Si: 1.739, P: 2.013, S: 2.307, Cl: 2.621, Ca: 3.69 (k eV)) (B). The total spectrum counts were 141821 in the EDS analysis, and the signals of Si, Al and Ca were mapped (C).
(PDF)

S9 Fig Effect of gluconic acid/gluconolactone removal on aluminum extraction capacity of *H*. *gigas* body fluid. Three *H*. *gigas* individuals were used for the experiment. We prepared *H*. *gigas* body fluid and removed gluconic acid/gluconolactone from *H*. *gigas* body fluid with enzymes as described in Materials and Methods (enzyme treated sample. Control sample was prepared without enzyme (control).
(PDF)

S10 Fig Extraction of iron from the sediment of Challenger Deep. The sediment was suspended in 25 mM sodium acetate buffer (pH 5.0) containing 10 mM sodium gluconic acid/gluconolactone or not (control). The suspension was pressurized at 100 MPa and incubated at 2°C for 1 h. After decompression, the sediment was separated with centrifugation (15,000 x *g* at 400B0C for 2 min). The iron content of the supernatant was measured as described in the Methods section. The error bar shows the S.D. (n = 3).
(PDF)

S11 Fig Effect of aluminum hydroxide gel on the release of protein from salmon roe under high pressure. Washed salmon roe were soaked and pressurized in artificial seawater at 100 MPa, which is the same pressure observed at approximately 10,000 m in depth, or in the control at 0.1 MPa, which is the same pressure observed in the atmosphere at sea level, for 24 h in a pressure-resistant bottle. The salmon roe that suffered the greatest damage after decompression are displayed in panel A. After decompression, the protein content of the artificial seawater was measured (panel B). The error bar shows the S.D. (n = 4). The bar in panel A indicates 1 cm.
(PDF)

S12 Fig Model of aluminum extraction and transport in *H*. *gigas*. *H*. *gigas* feeds on sea sediments as well as plant debris. The digestive enzyme cellulase produces glucose from the cellulose of plant debris, and then glucose 1-dehydrogenase produces gluconolactone from glucose. The gluconolactone extracts aluminum from the clay minerals in *H*. *gigas*’ gut, and the aluminum ions are transformed into aluminum hydroxide through contact with alkaline seawater, and they then adhere to the telson in their gel state. Gluconolactone also transforms into gluconic acid in alkaline seawater. Gluconic acid chelates aluminum ions and transports them throughout the entire exoskeleton. The red-colored chemicals and enzymes indicate the presence and reaction of aluminum in the gut, and the blue colored chemicals indicate the presence of aluminum in the exoskeleton with seawater.
(PDF)

S1 Table Amount of aluminum in the body of *H*. *gigas*. (DOCX)

S2 Table The TEM/EDS analysis of the sediments. (DOCX)

S3 Table Metabolic analysis of *H*. *gigas*. (DOCX)

S4 Table Amount of gluconic acid in the body of *H*. *gigas*. (DOCX)

S1 File Materials and methods of supporting Figs and Tables. (DOCX)
