# Supplementary figures and images for: An aluminum shield enables the amphipod Hirondellea gigas to inhabit deep-sea environments
Source: PLoS One. 2019 Apr 4;14(4):e0206710. doi: 10.1371/journal.pone.0206710 (PMC6449124; doi:10.1371/journal.pone.0206710)

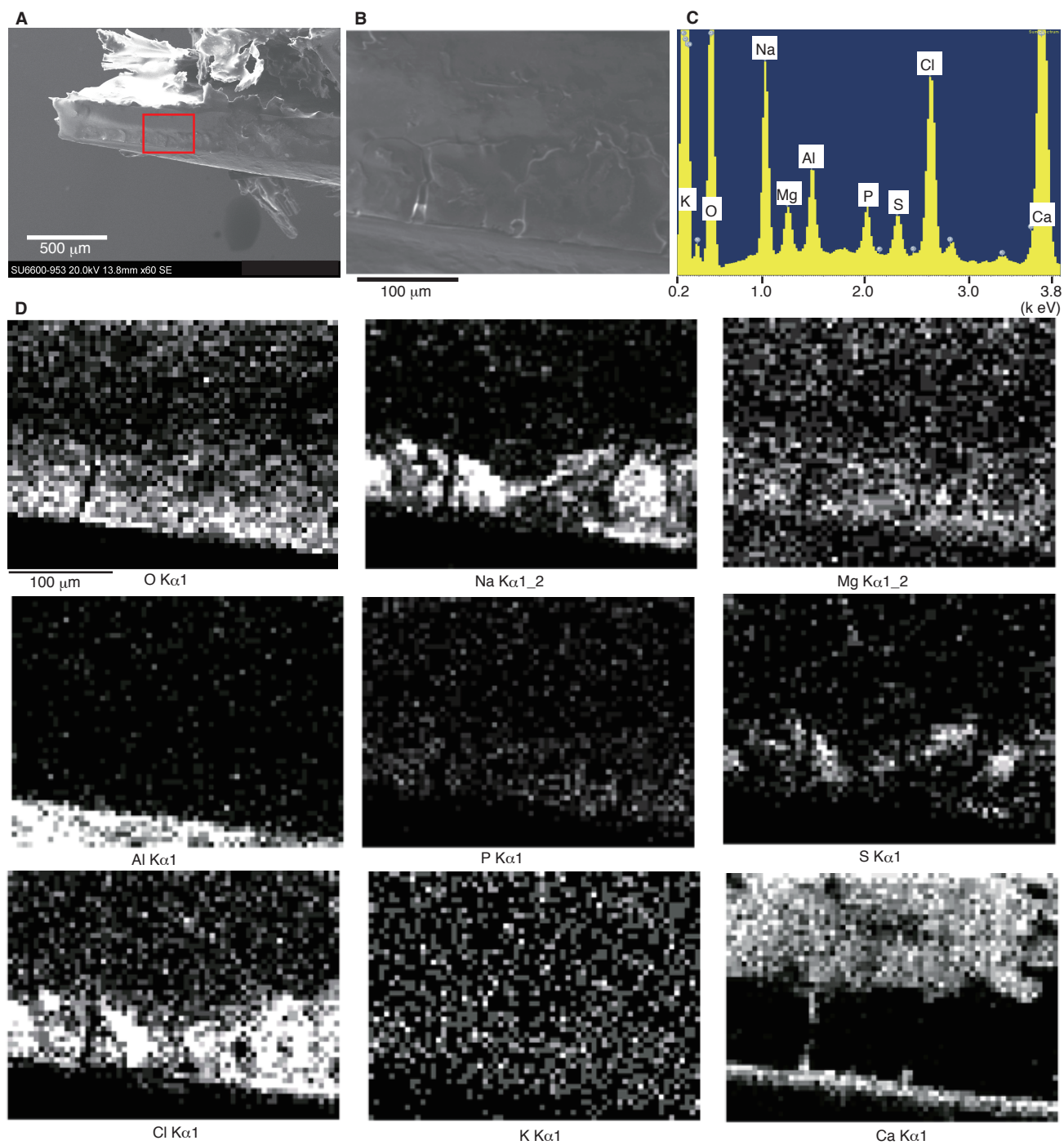

Supplement: S1 Fig — H. gigas specimens captured from Challenger Deep was freeze dried for SEM observations (A, B). Panel B shows an enlargement of the red square in panel A. SEM observations and EDS analyses were conducted without any coating. The EDS spectrum of panel B includes an annotation of each element with its Kα energy level (C: 0.284, O: 0.532, Na: 1.071, Mg: 1.253, Al: 1.486, P: 2.013, S: 2.307, Cl: 2.621, Ca: 3.69 (k eV)) (C). The total spectrum counts were 117,388 in the EDS analysis, and the major elements were mapped (D). (PDF) [file pone.0206710.s001.pdf]

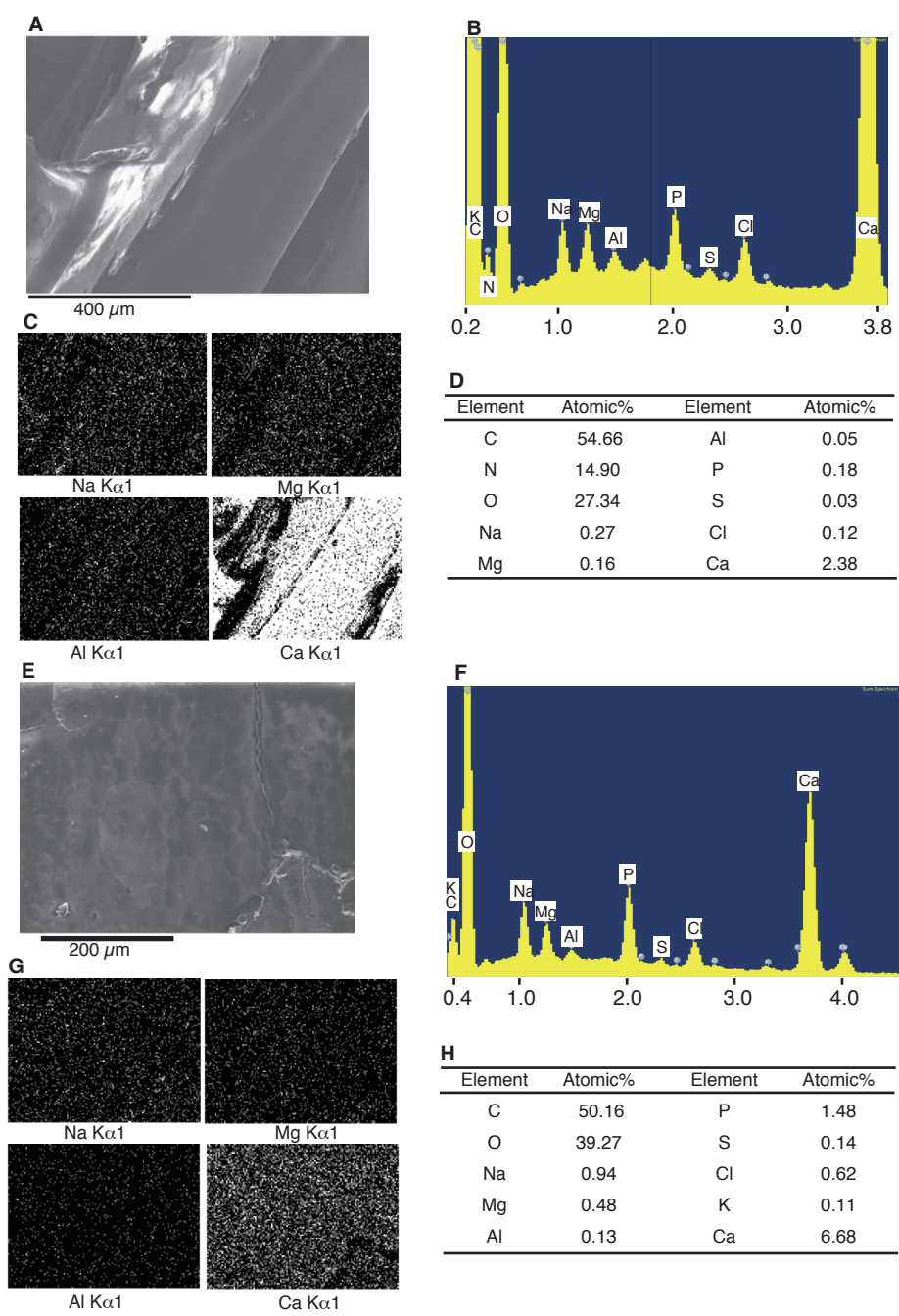

Supplement: S2 Fig — An SEM/EDS analysis was conducted on the telson region (A) and the exoskeleton (E) as described in the Methods section. The EDS spectra of panel A and E include annotations of each element with its Kα energy level (C: 0.284, O: 0.532, Na: 1.071, Mg: 1.253, Al: 1.486, P: 2.013, S: 2.307, Cl: 2.621, Ca: 3.69 (k eV)) (B, F). The metal peaks were mapped for the telson (C) and the exoskeleton (G). The composition of elements was calculated from the total spectrum counts (D: 320,357, H: 141,002). (PDF) [file pone.0206710.s002.pdf]

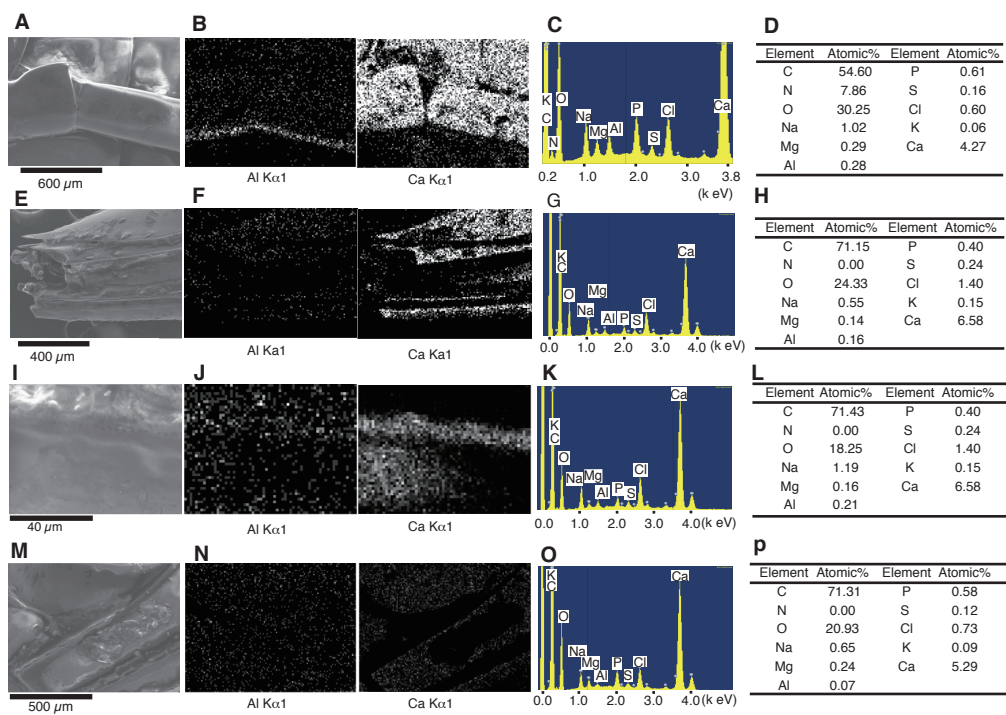

Supplement: S3 Fig — An SEM/EDS analysis was conducted on H. gigas captured from the Izu-Ogasawara Trench. Four views of the exoskeleton were analyzed (A, E, I, M) as described in the Methods section. Panels E, I, and M were observed with accelerating voltages of 15 kV, because oil components induce sample charging and cause drift in SEM/EDX images, which cannot be suppressed at high acceleration voltage sufficiently. Only calcium and aluminum were mapped (B, F, J, N). The EDS spectrum includes an annotation of each element with its Kα energy level (C: 0.284, O: 0.532, Na: 1.071, Mg: 1.253, Al: 1.486, P: 2.013, S: 2.307, Cl: 2.621, Ca: 3.69 (k eV)). All EDS signals were detected and calculated from the total spectrum counts (C and D, G and H, K and L, O and P). (PDF) [file pone.0206710.s003.pdf]

S4 Fig Kobayashi et al.

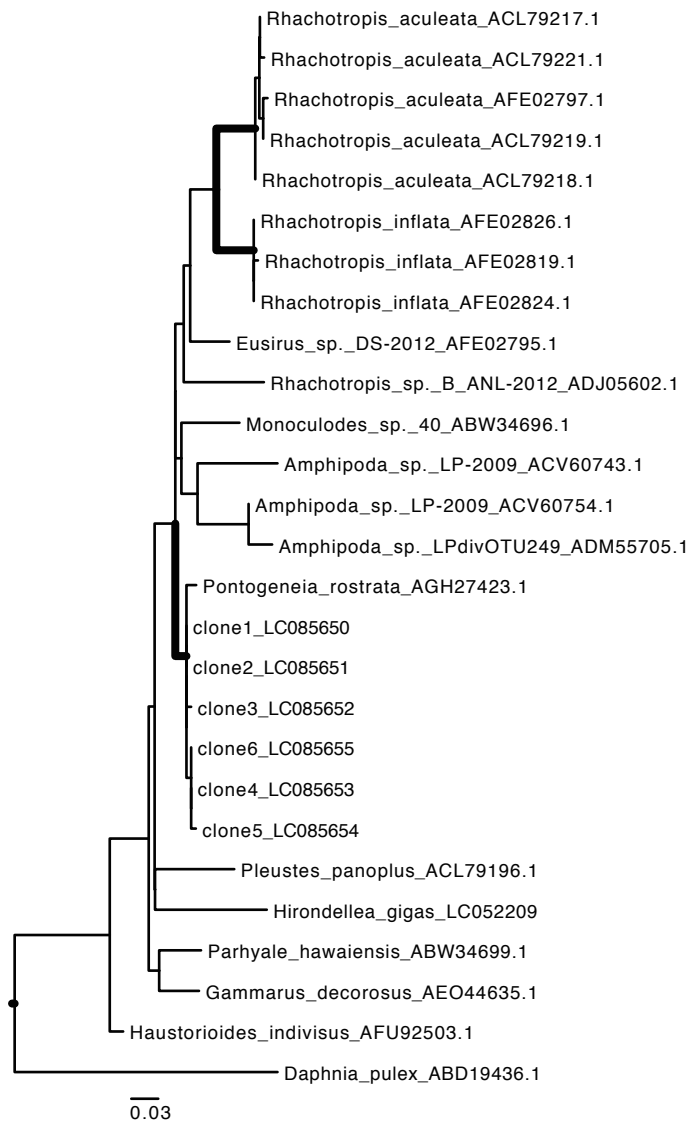

Supplement: S4 Fig — The COI genes were amplified and cloned in E. coli DH5α as described in the Methods section. Then, we decided DNA sequences of 6 E. coli clones. The amino acid sequence of the COI obtained from the coastal amphipods indicated “clone1-6” in this study. The amino acid sequences of the COI of related amphipods were obtained from GenBank, and each accession number was added after the species name. Bold lines indicate bootstrap support above 95% as inferred from the maximum likelihood analysis. The scale bar for the branch length is denoted by the estimated number of amino acid substitutions per site. (PDF) [file pone.0206710.s004.pdf]

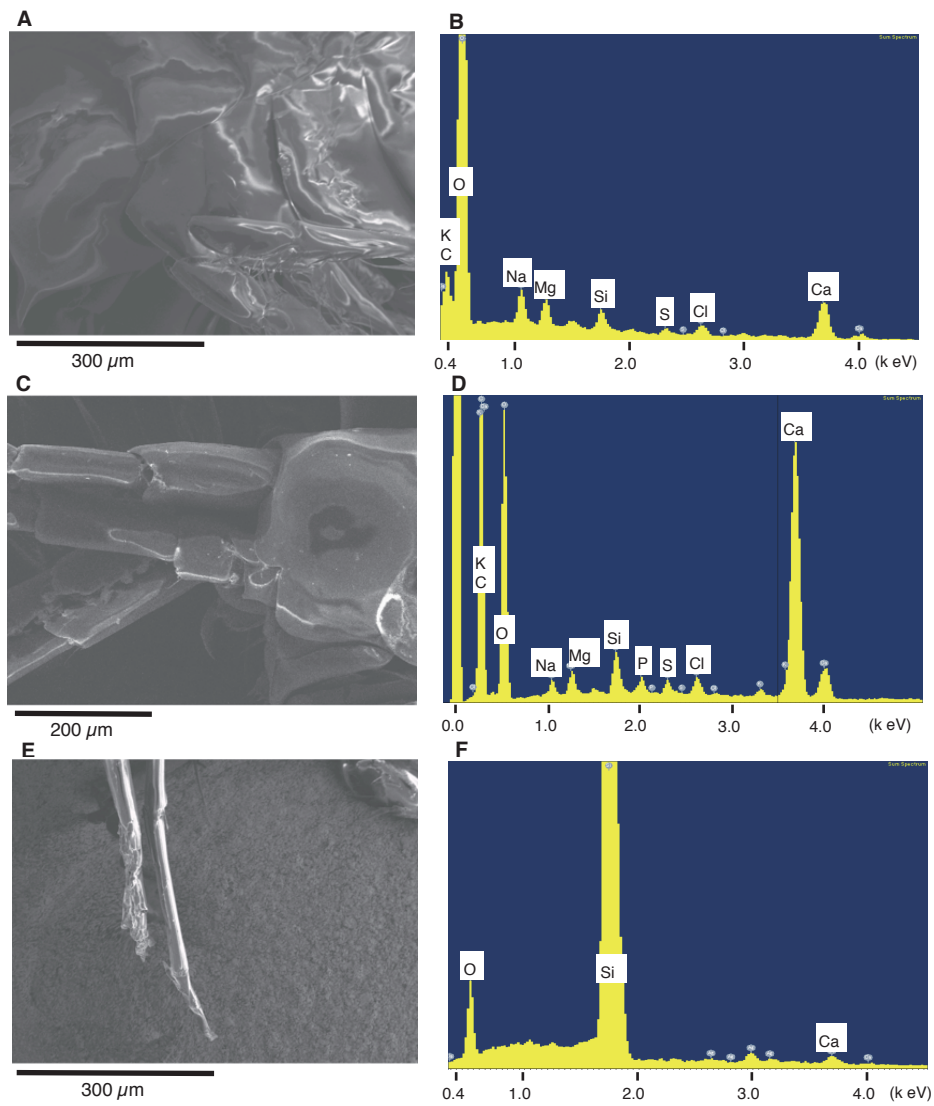

Supplement: S5 Fig — Three amphipods were freeze dried and then analyzed. The telson (A, C) and foot (E) of H. gigas contained aluminum. Panel A and B were observed with accelerating voltages of 10 kV because of sample movement related to the oil component. EDS analysis was conducted as described in the Methods section (B, D, F). The EDS spectrum includes an annotation of each element with its Kα energy level (C: 0.284, O: 0.532, Na: 1.071, Mg: 1.253, P: 2.013, S: 2.307, Cl: 2.621, Ca: 3.69 (k eV)). The peak of Si was obtained from the backfield in F. (PDF) [file pone.0206710.s005.pdf]

S6 Fig Kobayashi et al.

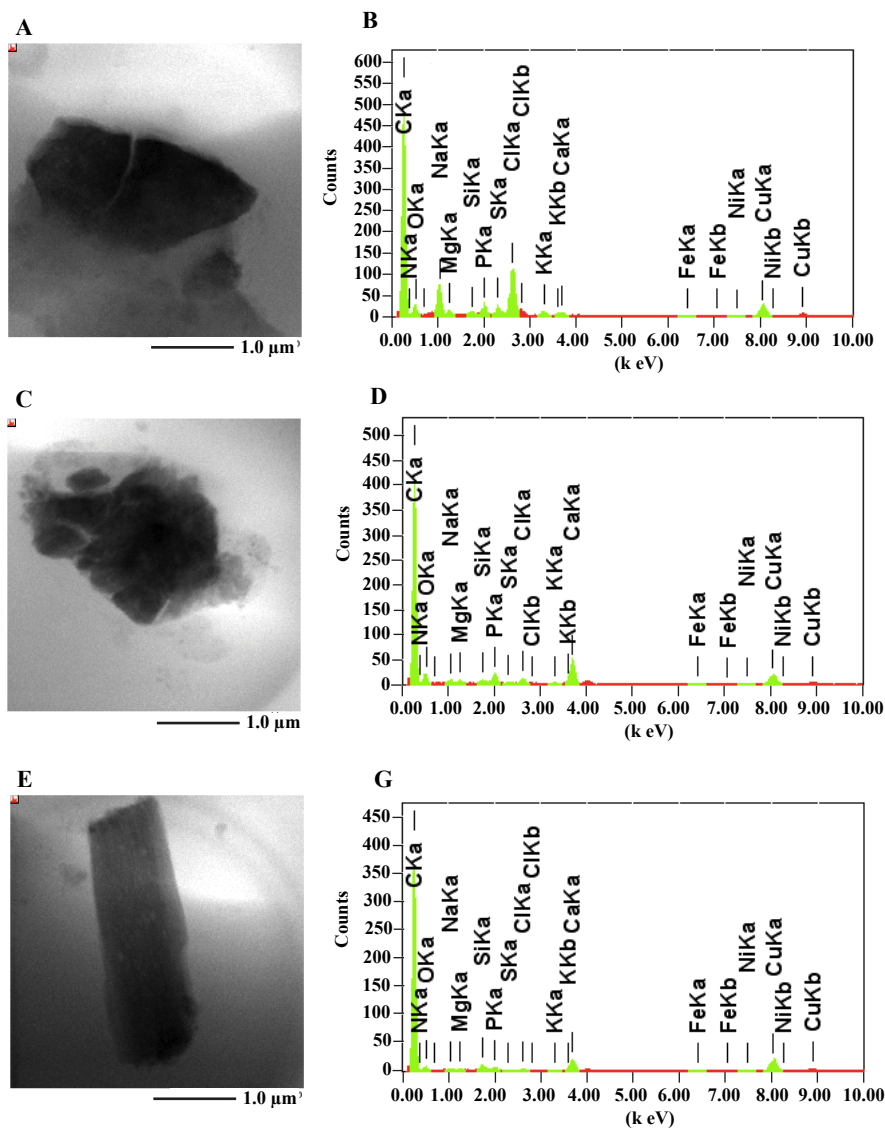

Supplement: S6 Fig — Exoskeletons of the amphipods captured from the Izu-Ogasawara Trench were removed from the individuals, freeze dried, and then scrapped. Bright-field STEM observations were conducted for pieces of the exoskeletons (A, C, E). Characteristic X-rays were collected over 60 s (B, D, F). The Cu or Mo signals were caused by the TEM grid. The Si signal was background. (PDF) [file pone.0206710.s006.pdf]

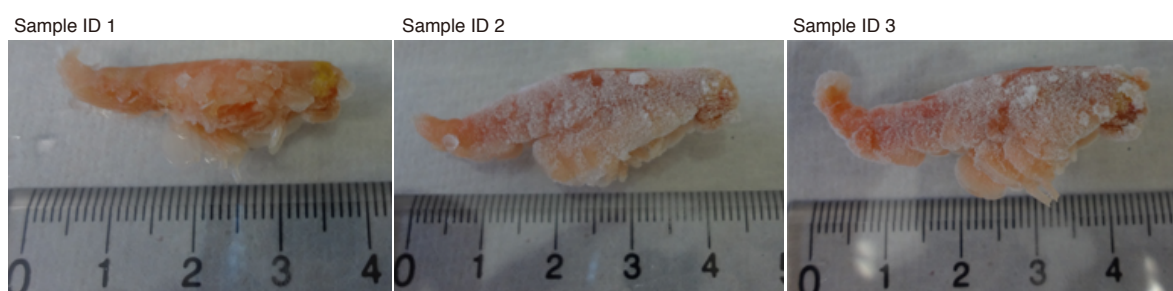

Supplement: S7 Fig — Deep-sea amphipod H. gigas individuals were immediately frozen and maintained at -80°C after capture from Challenger Deep. These amphipods were selected randomly from frozen stock. (PDF) [file pone.0206710.s007.pdf]

S8 Fig Kobayashi et al.

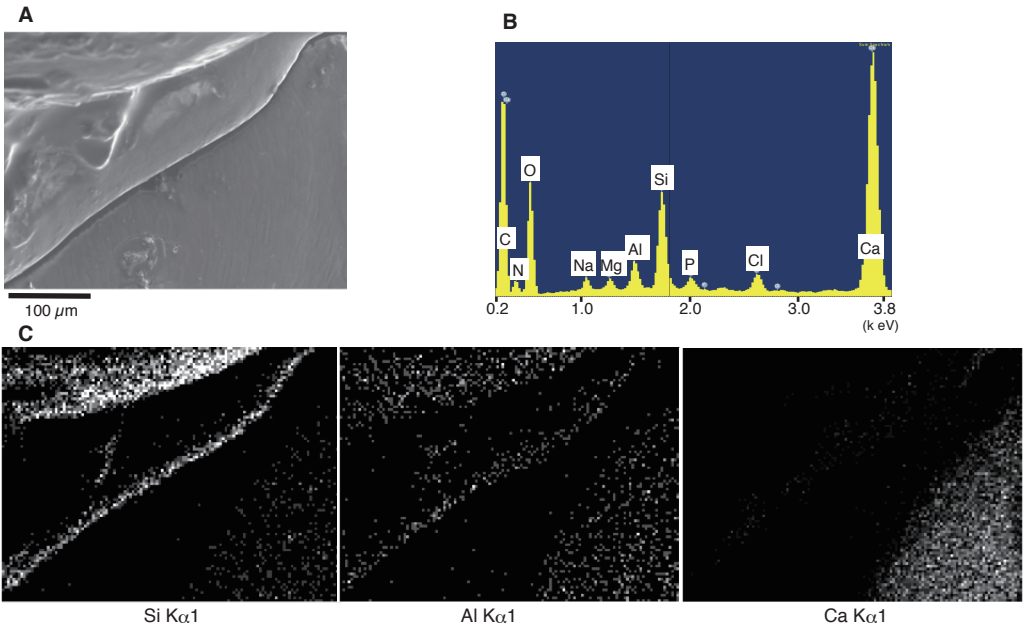

Supplement: S8 Fig — H. gigas specimens captured from Challenger Deep were freeze dried for the SEM observations (A). SEM observations were conducted without any coating. The EDS spectrum included an annotation of each element with its Kα energy level (C: 0.284, O: 0.532, Na: 1.071, Mg: 1.253, Al: 1.486, Si: 1.739, P: 2.013, S: 2.307, Cl: 2.621, Ca: 3.69 (k eV)) (B). The total spectrum counts were 141821 in the EDS analysis, and the signals of Si, Al and Ca were mapped (C). (PDF) [file pone.0206710.s008.pdf]

S9 Fig Kobayashi et al.

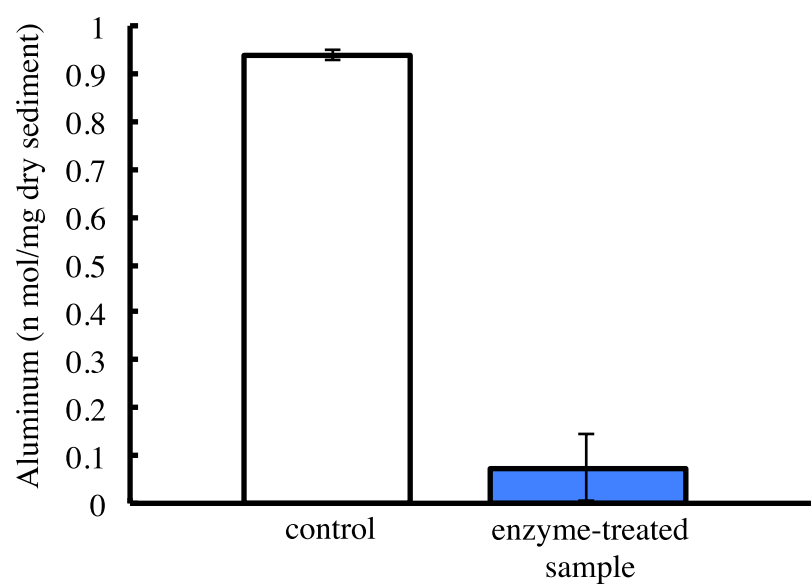

Supplement: S9 Fig — Three H. gigas individuals were used for the experiment. We prepared H. gigas body fluid and removed gluconic acid/gluconolactone from H. gigas body fluid with enzymes as described in Materials and Methods (enzyme treated sample. Control sample was prepared without enzyme (control). (PDF) [file pone.0206710.s009.pdf]

S10 Fig Kobayashi et al.

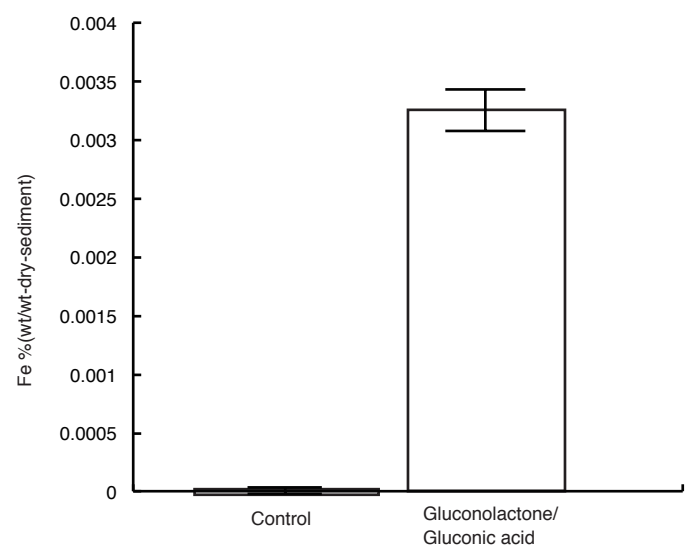

Supplement: S10 Fig — The sediment was suspended in 25 mM sodium acetate buffer (pH 5.0) containing 10 mM sodium gluconic acid/gluconolactone or not (control). The suspension was pressurized at 100 MPa and incubated at 2°C for 1 h. After decompression, the sediment was separated with centrifugation (15,000 x g at 400B0C for 2 min). The iron content of the supernatant was measured as described in the Methods section. The error bar shows the S.D. (n = 3). (PDF) [file pone.0206710.s010.pdf]

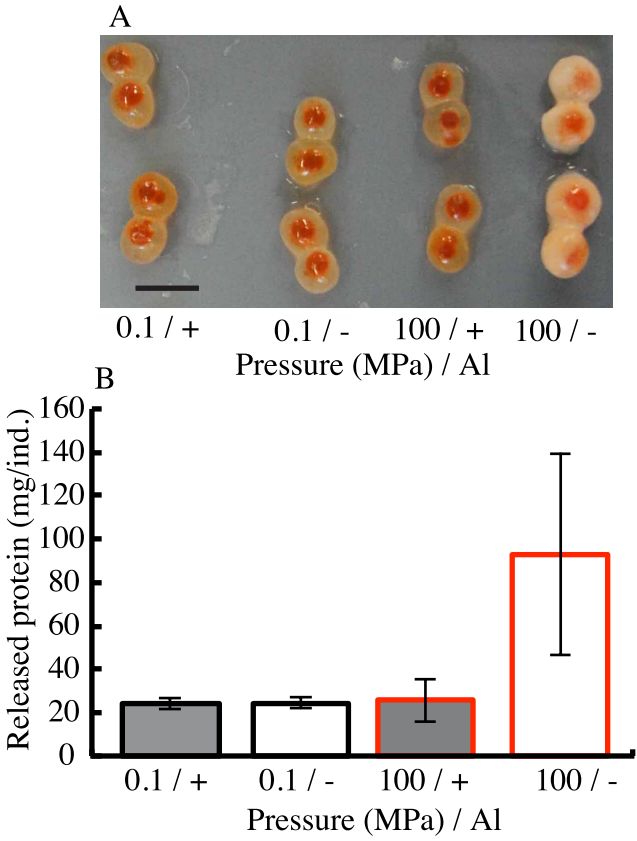

Supplement: S11 Fig — Washed salmon roe were soaked and pressurized in artificial seawater at 100 MPa, which is the same pressure observed at approximately 10,000 m in depth, or in the control at 0.1 MPa, which is the same pressure observed in the atmosphere at sea level, for 24 h in a pressure-resistant bottle. The salmon roe that suffered the greatest damage after decompression are displayed in panel A. After decompression, the protein content of the artificial seawater was measured (panel B). The error bar shows the S.D. (n = 4). The bar in panel A indicates 1 cm. (PDF) [file pone.0206710.s011.pdf]

S12 Figure Kobayashi et al.

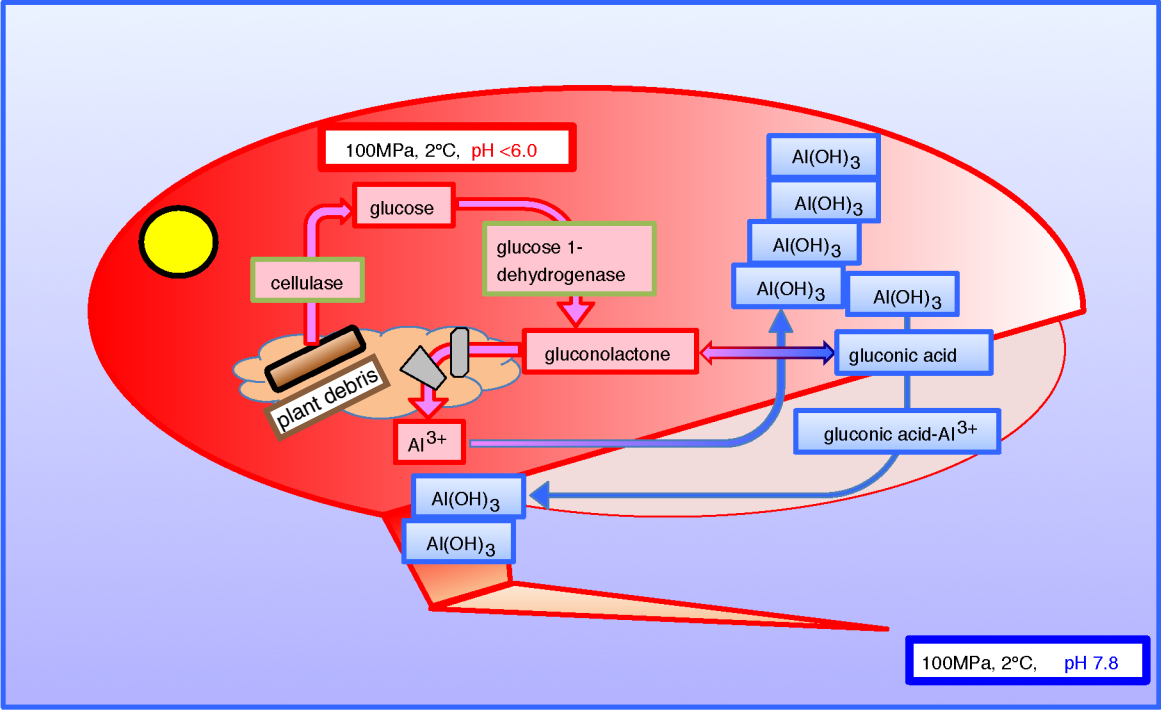

Supplement: S12 Fig — H. gigas feeds on sea sediments as well as plant debris. The digestive enzyme cellulase produces glucose from the cellulose of plant debris, and then glucose 1-dehydrogenase produces gluconolactone from glucose. The gluconolactone extracts aluminum from the clay minerals in H. gigas’ gut, and the aluminum ions are transformed into aluminum hydroxide through contact with alkaline seawater, and they then adhere to the telson in their gel state. Gluconolactone also transforms into gluconic acid in alkaline seawater. Gluconic acid chelates aluminum ions and transports them throughout the entire exoskeleton. The red-colored chemicals and enzymes indicate the presence and reaction of aluminum in the gut, and the blue colored chemicals indicate the presence of aluminum in the exoskeleton with seawater. (PDF) [file pone.0206710.s012.pdf]
