# Supplementary material for: An aluminum shield enables the amphipod Hirondellea gigas to inhabit deep-sea environments
Source: PLoS One. 2019 Apr 4;14(4):e0206710. doi: 10.1371/journal.pone.0206710 (PMC6449124; doi:10.1371/journal.pone.0206710)
Supplement: S1 Table — (DOCX) [file pone.0206710.s013.docx]

S1 Table Amount of aluminum in the body of *H. gigas*

| Sample ID | Body fluid (µ mol) | | Exoskeleton  (µ mol) | Total  (µ mol) |
| --- | --- | --- | --- | --- |
|  | Water-phase | Lipid-phase (x10^-3^) |  |  |
| 1 | 0.255 ± 0.059 | 3.14 ± 1.7 | 1.05 ± 0.16 | 1.31 ± 0.22 |
| 2 | 0.827 ± 0.017 | 4.19 ± 0.32 | 1.29 ± 0.15 | 2.12 ± 0.17 |
| 3 | 1.16 ± 0.13 | 23.9 ± 6.8 | 1.18 ± 0.10 | 2.36 ± 0.24 |
