# Supplementary material for: An aluminum shield enables the amphipod Hirondellea gigas to inhabit deep-sea environments
Source: PLoS One. 2019 Apr 4;14(4):e0206710. doi: 10.1371/journal.pone.0206710 (PMC6449124; doi:10.1371/journal.pone.0206710)
Supplement: S2 Table — (DOCX) [file pone.0206710.s014.docx]

S2 Table The TEM/EDS analysis of the sediments

|  | Sample ID | | | | | |
| --- | --- | --- | --- | --- | --- | --- |
| Element | #1 | #2 | #3 | #4 | #5 | #6 |
| C (standaed) | 66.07 | 54.12 | 72.49 | 63.84 | 74.13 | 71.41 |
| O | 6.55 | 9.51 | 4.57 | 8.52 | 5.07 | 6 |
| Mg | 2.13 | 3.24 | 1.34 | 2.02 | 1.99 | 0.77 |
| Al | 1.23 | 2.43 | 1.13 | 1.69 | 0.99 | 1.14 |
| Si | 5.6 | 8.72 | 3.77 | 6.71 | 3.6 | 5.66 |
| K | 0.15 | 0.3 | 0.2 | 0.18 | 0.19 | 0.33 |
| Fe | 2.44 | 2.99 | 1.93 | 3.7 | 0.97 | 2.5 |
| Cu^1)^ | 15.82 | 18.68 | 14.56 | 13.34 | 13.06 | 12.2 |
| Total | 99.99 | 99.99 | 99.99 | 100 | 100 | 100.01 |
| Al:Si | 4.55 | 3.59 | 3.34 | 3.97 | 3.64 | 4.96 |

Weight (%)

1) Background
