# Supplementary material for: An aluminum shield enables the amphipod Hirondellea gigas to inhabit deep-sea environments
Source: PLoS One. 2019 Apr 4;14(4):e0206710. doi: 10.1371/journal.pone.0206710 (PMC6449124; doi:10.1371/journal.pone.0206710)
Supplement: S3 Table — (DOCX) [file pone.0206710.s015.docx]

S3 Table Metabolic analysis of *H. gigas*

| Compound name | KEGG ID^1)^ |
| --- | --- |
|  |  |
| Lactic acid | [C00186, C00256, C01432](http://www.genome.jp/dbget-bin/www_bget?cpd:C00186) |
| 2-Hydroxybutyric acid | [C05984](http://www.genome.jp/dbget-bin/www_bget?cpd:C05984) |
| Glyceric acid | [C00258](http://www.genome.jp/dbget-bin/www_bget?cpd:C00258) |
| Succinic acid | [C00042](http://www.genome.jp/dbget-bin/www_bget?cpd:C00042) |
| **2-Aminoethylphosphonic acid**^2)^ | [C03557](http://www.genome.jp/dbget-bin/www_bget?cpd:C03557) |
| **4-Methyl-2-oxovaleric acid 3-Methyl-2-oxovaleric acid** | [C00233 C00671, C03465](http://www.genome.jp/dbget-bin/www_bget?cpd:C00233) |
| Malic acid | [C00149, C00497, C00711](http://www.genome.jp/dbget-bin/www_bget?cpd:C00149) |
| **2-Hydroxyglutaric acid** | [C02630, C01087, C03196](http://www.genome.jp/dbget-bin/www_bget?cpd:C02630) |
| Xanthine | [C00385](http://www.genome.jp/dbget-bin/www_bget?cpd:C00385) |
| **Pelargonic acid** | [C01601](http://www.genome.jp/dbget-bin/www_bget?cpd:C01601) |
| Uric acid | [C00366](http://www.genome.jp/dbget-bin/www_bget?cpd:C00366) |
| Dihydroxyacetone phosphate | [C00111](http://www.genome.jp/dbget-bin/www_bget?cpd:C00111) |
| Glycerol 3-phosphate | [C00093](http://www.genome.jp/dbget-bin/www_bget?cpd:C00093) |
| 3-Phosphoglyceric acid | [C00197](http://www.genome.jp/dbget-bin/www_bget?cpd:C00197) |
| Citric acid | [C00158](http://www.genome.jp/dbget-bin/www_bget?cpd:C00158) |
| **Gluconic acid** | [C00257](http://www.genome.jp/dbget-bin/www_bget?cpd:C00257) |
| **Lauric acid** | [C02679](http://www.genome.jp/dbget-bin/www_bget?cpd:C02679) |
| Xanthurenic acid | [C02470](http://www.genome.jp/dbget-bin/www_bget?cpd:C02470) |
| Ribulose 5-phosphate | [C00199, C01101](http://www.genome.jp/dbget-bin/www_bget?cpd:C00199) |
| XA0033^3)^ | No ID |
| ***myo*-Inositol 1-phosphate *myo*-Inositol 3-phosphate** | [C01177 C04006](http://www.genome.jp/dbget-bin/www_bget?cpd:C01177) |
| Fructose 6-phosphate | [C05345, C00085](http://www.genome.jp/dbget-bin/www_bget?cpd:C05345) |
| Glucose 6-phosphate | [C00668, C01172, C00092](http://www.genome.jp/dbget-bin/www_bget?cpd:C00668) |
| Glucose 1-phosphate | [C00103](http://www.genome.jp/dbget-bin/www_bget?cpd:C00103) |
| Sedoheptulose 7-phosphate | [C05382](http://www.genome.jp/dbget-bin/www_bget?cpd:C05382) |
| 3'-CMP **2'-CMP** | [C05822 C03104](http://www.genome.jp/dbget-bin/www_bget?cpd:C05822) |
| cAMP | [C00575](http://www.genome.jp/dbget-bin/www_bget?cpd:C00575) |
| Fructose 1,6-diphosphate | [C00354](http://www.genome.jp/dbget-bin/www_bget?cpd:C00354) |
| AMP | [C00020](http://www.genome.jp/dbget-bin/www_bget?cpd:C00020) |
| **3'-AMP** | [C01367](http://www.genome.jp/dbget-bin/www_bget?cpd:C01367) |
| IMP | [C00130](http://www.genome.jp/dbget-bin/www_bget?cpd:C00130) |
| GMP | [C00144](http://www.genome.jp/dbget-bin/www_bget?cpd:C00144) |
| Trehalose 6-phosphate | [C00689](http://www.genome.jp/dbget-bin/www_bget?cpd:C00689) |
| ADP | [C00008](http://www.genome.jp/dbget-bin/www_bget?cpd:C00008) |
| ATP | [C00002](http://www.genome.jp/dbget-bin/www_bget?cpd:C00002) |
| Taurocholic acid | [C05122](http://www.genome.jp/dbget-bin/www_bget?cpd:C05122) |
| UDP-*N*-acetylglucosamine | [C00043](http://www.genome.jp/dbget-bin/www_bget?cpd:C00043) |
| NAD^+^ | [C00003](http://www.genome.jp/dbget-bin/www_bget?cpd:C00003) |
| Trimethylamine | [C00565](http://www.genome.jp/dbget-bin/www_bget?cpd:C00565) |
| **Ethanolamine** | [C00189](http://www.genome.jp/dbget-bin/www_bget?cpd:C00189) |
| Gly | [C00037](http://www.genome.jp/dbget-bin/www_bget?cpd:C00037) |
| β-Ala | [C00099](http://www.genome.jp/dbget-bin/www_bget?cpd:C00099) |
| Sarcosine | [C00213](http://www.genome.jp/dbget-bin/www_bget?cpd:C00213) |
| Ala | [C00041, C00133, C01401](http://www.genome.jp/dbget-bin/www_bget?cpd:C00041) |
| **Glycerol** | [C00116](http://www.genome.jp/dbget-bin/www_bget?cpd:C00116) |
| **Azetidine 2-carboxylic acid** | [C08267](http://www.genome.jp/dbget-bin/www_bget?cpd:C08267) |
| **3-Aminoisobutyric acid** | [C03284, C05145](http://www.genome.jp/dbget-bin/www_bget?cpd:C03284) |
| **2-Aminobutyric acid** | [C02261, C02356](http://www.genome.jp/dbget-bin/www_bget?cpd:C02261) |
| GABA | [C00334](http://www.genome.jp/dbget-bin/www_bget?cpd:C00334) |
| *N*,*N*-Dimethylglycine | [C01026](http://www.genome.jp/dbget-bin/www_bget?cpd:C01026) |
| 3-Aminobutyric acid | No ID |
| Choline | [C00114](http://www.genome.jp/dbget-bin/www_bget?cpd:C00114) |
| Ser | [C00065, C00716, C00740](http://www.genome.jp/dbget-bin/www_bget?cpd:C00065) |
| Hypotaurine | [C00519](http://www.genome.jp/dbget-bin/www_bget?cpd:C00519) |
| Histamine | [C00388](http://www.genome.jp/dbget-bin/www_bget?cpd:C00388) |
| Uracil | [C00106](http://www.genome.jp/dbget-bin/www_bget?cpd:C00106) |
| Creatinine | [C00791](http://www.genome.jp/dbget-bin/www_bget?cpd:C00791) |
| Pro | [C00148, C00763, C16435](http://www.genome.jp/dbget-bin/www_bget?cpd:C00148) |
| Betaine | [C00719](http://www.genome.jp/dbget-bin/www_bget?cpd:C00719) |
| Val | [C00183, C06417, C16436](http://www.genome.jp/dbget-bin/www_bget?cpd:C00183) |
| **5-Aminovaleric acid** | [C00431](http://www.genome.jp/dbget-bin/www_bget?cpd:C00431) |
| **2-Methylserine** | [C02115](http://www.genome.jp/dbget-bin/www_bget?cpd:C02115) |
| Thr | [C00188, C00820](http://www.genome.jp/dbget-bin/www_bget?cpd:C00188) |
| Cys | [C00097, C00736, C00793](http://www.genome.jp/dbget-bin/www_bget?cpd:C00097) |
| Nicotinic acid | [C00253](http://www.genome.jp/dbget-bin/www_bget?cpd:C00253) |
| Taurine | [C00245](http://www.genome.jp/dbget-bin/www_bget?cpd:C00245) |
| Thymine | [C00178](http://www.genome.jp/dbget-bin/www_bget?cpd:C00178) |
| Imidazole-4-acetic acid | [C02835](http://www.genome.jp/dbget-bin/www_bget?cpd:C02835) |
| XC0016^3)^ | No ID |
| ***N*-Methylproline** | No ID |
| *trans*-Glutaconic acid | [C02214](http://www.genome.jp/dbget-bin/www_bget?cpd:C02214) |
| *cis*-4-Hydroxyproline | [C03440](http://www.genome.jp/dbget-bin/www_bget?cpd:C03440) |
| Hydroxyproline | [C01157](http://www.genome.jp/dbget-bin/www_bget?cpd:C01157) |
| Creatine | [C00300](http://www.genome.jp/dbget-bin/www_bget?cpd:C00300) |
| Ile | [C00407, C06418, C16434](http://www.genome.jp/dbget-bin/www_bget?cpd:C00407) |
| Leu | [C00123, C01570, C16439](http://www.genome.jp/dbget-bin/www_bget?cpd:C00123) |
| **Norspermidine** | [C03375](http://www.genome.jp/dbget-bin/www_bget?cpd:C03375) |
| **Gly-Gly** | [C02037](http://www.genome.jp/dbget-bin/www_bget?cpd:C02037) |
| Asn | [C00152, C01905, C16438](http://www.genome.jp/dbget-bin/www_bget?cpd:C00152) |
| Ornithine | [C00077, C00515, C01602](http://www.genome.jp/dbget-bin/www_bget?cpd:C00077) |
| Thiaproline | No ID |
| Asp | [C00049, C00402, C16433](http://www.genome.jp/dbget-bin/www_bget?cpd:C00049) |
| Adenine | [C00147](http://www.genome.jp/dbget-bin/www_bget?cpd:C00147) |
| Hypoxanthine | [C00262](http://www.genome.jp/dbget-bin/www_bget?cpd:C00262) |
| Trigonelline | [C01004](http://www.genome.jp/dbget-bin/www_bget?cpd:C01004) |
| Stachydrine | [C10172](http://www.genome.jp/dbget-bin/www_bget?cpd:C10172) |
| γ-Butyrobetaine | [C01181](http://www.genome.jp/dbget-bin/www_bget?cpd:C01181) |
| Spermidine | [C00315](http://www.genome.jp/dbget-bin/www_bget?cpd:C00315) |
| Gln | [C00064, C00303, C00819](http://www.genome.jp/dbget-bin/www_bget?cpd:C00064) |
| Lys | [C00047, C00739, C16440](http://www.genome.jp/dbget-bin/www_bget?cpd:C00047) |
| *threo*-β-Methylaspartic acid | [C03618](http://www.genome.jp/dbget-bin/www_bget?cpd:C03618) |
| Glu | [C00025, C00217, C00302](http://www.genome.jp/dbget-bin/www_bget?cpd:C00025) |
| Met | [C00073, C00855, C01733](http://www.genome.jp/dbget-bin/www_bget?cpd:C00073) |
| Triethanolamine | [C06771](http://www.genome.jp/dbget-bin/www_bget?cpd:C06771) |
| Guanine | [C00242](http://www.genome.jp/dbget-bin/www_bget?cpd:C00242) |
| His | [C00135, C00768, C06419](http://www.genome.jp/dbget-bin/www_bget?cpd:C00135) |
| **Betonicine** | [C08269](http://www.genome.jp/dbget-bin/www_bget?cpd:C08269) |
| **Ala-Ala** | [C00993](http://www.genome.jp/dbget-bin/www_bget?cpd:C00993) |
| 2-Aminoadipic acid | [C00956](http://www.genome.jp/dbget-bin/www_bget?cpd:C00956) |
| Carnitine | [C00318, C00487, C15025](http://www.genome.jp/dbget-bin/www_bget?cpd:C00318) |
| Phe | [C00079, C02057, C02265](http://www.genome.jp/dbget-bin/www_bget?cpd:C00079) |
| Pyridoxamine | [C00534](http://www.genome.jp/dbget-bin/www_bget?cpd:C00534) |
| Tyr-Arg_divalent | No ID |
| 3-Methylhistidine | [C01152](http://www.genome.jp/dbget-bin/www_bget?cpd:C01152) |
| ***N*^5^-Ethylglutamine** | [C01047](http://www.genome.jp/dbget-bin/www_bget?cpd:C01047) |
| *N*-Acetylornithine | [C00437](http://www.genome.jp/dbget-bin/www_bget?cpd:C00437) |
| Citrulline | [C00327](http://www.genome.jp/dbget-bin/www_bget?cpd:C00327) |
| Arg | [C00062, C00792](http://www.genome.jp/dbget-bin/www_bget?cpd:C00062) |
| Glucosamine | [C00329](http://www.genome.jp/dbget-bin/www_bget?cpd:C00329) |
| Tyr | [C00082, C01536, C06420](http://www.genome.jp/dbget-bin/www_bget?cpd:C00082) |
| **Phosphorylcholine** | [C00588](http://www.genome.jp/dbget-bin/www_bget?cpd:C00588) |
| **Gly-Leu** | No ID |
| ***N*^6^-Acetyllysine** | [C02727](http://www.genome.jp/dbget-bin/www_bget?cpd:C02727) |
| *N*^6^,*N*^6^,*N*^6^-Trimethyllysine | [C03793](http://www.genome.jp/dbget-bin/www_bget?cpd:C03793) |
| **Homocitrulline** | [C02427](http://www.genome.jp/dbget-bin/www_bget?cpd:C02427) |
| **Gly-Asp** | No ID |
| **2,6-Diaminopimelic acid** | [C00666](http://www.genome.jp/dbget-bin/www_bget?cpd:C00666) |
| ***N*-Acetylhistidine** | [C02997](http://www.genome.jp/dbget-bin/www_bget?cpd:C02997) |
| **N,N-Dimethylarginine** | [C03626](http://www.genome.jp/dbget-bin/www_bget?cpd:C03626) |
| *O*-Acetylcarnitine | [C02571](http://www.genome.jp/dbget-bin/www_bget?cpd:C02571) |
| Trp | [C00078, C00525, C00806](http://www.genome.jp/dbget-bin/www_bget?cpd:C00078) |
| Kynurenine | [C00328, C01718](http://www.genome.jp/dbget-bin/www_bget?cpd:C00328) |
| **3-Methoxytyrosine** | No ID |
| XC0061^3)^ | No ID |
| **β-Ala-Lys** | [C05341](http://www.genome.jp/dbget-bin/www_bget?cpd:C05341) |
| ***N*-Acetylgalactosamine** | [C01132](http://www.genome.jp/dbget-bin/www_bget?cpd:C01132) |
| 3-Hydroxykynurenine | No ID |
| **γ-Glu-2-aminobutyric acid** | No ID |
| Ser-Glu | No ID |
| Cystine | [C00491, C01420](http://www.genome.jp/dbget-bin/www_bget?cpd:C00491) |
| Thymidine | [C00214](http://www.genome.jp/dbget-bin/www_bget?cpd:C00214) |
| Cytidine | [C00475](http://www.genome.jp/dbget-bin/www_bget?cpd:C00475) |
| Uridine | [C00299](http://www.genome.jp/dbget-bin/www_bget?cpd:C00299) |
| **Pyridoxamine 5'-phosphate** | [C00647](http://www.genome.jp/dbget-bin/www_bget?cpd:C00647) |
| γ-Glu-Cys | [C00669](http://www.genome.jp/dbget-bin/www_bget?cpd:C00669) |
| XC0089^3)^ | No ID |
| Glycerophosphocholine | [C00670](http://www.genome.jp/dbget-bin/www_bget?cpd:C00670) |
| Adenosine | [C00212](http://www.genome.jp/dbget-bin/www_bget?cpd:C00212) |
| 2'-Deoxyguanosine | [C00330](http://www.genome.jp/dbget-bin/www_bget?cpd:C00330) |
| Inosine | [C00294](http://www.genome.jp/dbget-bin/www_bget?cpd:C00294) |
| **Glu-Glu** | [C01425](http://www.genome.jp/dbget-bin/www_bget?cpd:C01425) |
| Saccharopine | [C00449](http://www.genome.jp/dbget-bin/www_bget?cpd:C00449) |
| Guanosine | [C00387](http://www.genome.jp/dbget-bin/www_bget?cpd:C00387) |
| **His-Glu** | No ID |
| Argininosuccinic acid | [C03406](http://www.genome.jp/dbget-bin/www_bget?cpd:C03406) |
| **Arg-Glu** | No ID |
| Glutathione (GSSG)_divalent | [C00127](http://www.genome.jp/dbget-bin/www_bget?cpd:C00127) |
| Glutathione (GSH) | [C00051](http://www.genome.jp/dbget-bin/www_bget?cpd:C00051) |
| Tyr-Glu | No ID |
| NMN | [C00455](http://www.genome.jp/dbget-bin/www_bget?cpd:C00455) |
| *S*-Adenosylhomocysteine | [C00021](http://www.genome.jp/dbget-bin/www_bget?cpd:C00021) |
| *S*-Adenosylmethionine | [C00019](http://www.genome.jp/dbget-bin/www_bget?cpd:C00019) |
| **Cysteine glutathione disulfide** | [C05526](http://www.genome.jp/dbget-bin/www_bget?cpd:C05526) |
| Myristic acid | [C06424](http://www.genome.jp/dbget-bin/www_bget?cpd:C06424) |
| Palmitoleic acid | [C08362](http://www.genome.jp/dbget-bin/www_bget?cpd:C08362) |
| Palmitic acid | [C00249](http://www.genome.jp/dbget-bin/www_bget?cpd:C00249) |
| Fatty acid (17:1) | No ID |
| Fatty acid (17:0) | No ID |
| Heptadecanoic acid | No ID |
| Stearidonic acid | [C16300](http://www.genome.jp/dbget-bin/www_bget?cpd:C16300) |
| Linolenic acid | [C06427](http://www.genome.jp/dbget-bin/www_bget?cpd:C06427) |
| Linoleic acid | [C01595](http://www.genome.jp/dbget-bin/www_bget?cpd:C01595) |
| Oleic acid | [C00712](http://www.genome.jp/dbget-bin/www_bget?cpd:C00712) |
| Stearic acid | [C01530](http://www.genome.jp/dbget-bin/www_bget?cpd:C01530) |
| Fatty acid (19:1) | No ID |
| *cis*-5,8,11,14,17-Eicosapentaenoic acid | [C06428](http://www.genome.jp/dbget-bin/www_bget?cpd:C06428) |
| Arachidonic acid | [C00219](http://www.genome.jp/dbget-bin/www_bget?cpd:C00219) |
| *cis*-8,11,14-Eicosatrienoic acid | [C03242](http://www.genome.jp/dbget-bin/www_bget?cpd:C03242) |
| *cis*-11,14-Eicosadienoic acid | [C16525](http://www.genome.jp/dbget-bin/www_bget?cpd:C16525) |
| ***cis*-11-Eicosenoic acid *cis*-13-Eicosenoic acid** | [C16526 No ID](http://www.genome.jp/dbget-bin/www_bget?cpd:C16526) |
| **15(S)-** **Hydroxyeicosatetraenoic acid** | [C04742](http://www.genome.jp/dbget-bin/www_bget?cpd:C04742) |
| *cis*-4,7,10,13,16,19-Docosahexaenoic acid | [C06429](http://www.genome.jp/dbget-bin/www_bget?cpd:C06429) |
| Fatty acid (22:5) | No ID |
| Fatty acid (22:4) | No ID |
| Fatty acid (22:2) | No ID |
| **Fatty acid (22:1) Erucic acid** | [No ID C08316](http://www.genome.jp/dbget-bin/www_bget?cpd:C08316) |
| **Prostaglandin D2** | [C00696](http://www.genome.jp/dbget-bin/www_bget?cpd:C00696) |
| **Prostaglandin D1** | [C06438](http://www.genome.jp/dbget-bin/www_bget?cpd:C06438) |
| Fatty acid (24:5) | No ID |
| Nervonic acid | [C08323](http://www.genome.jp/dbget-bin/www_bget?cpd:C08323) |
| **1-Palmitoyl-glycero-3-phosphoethanolamine** | No ID |
| **7-Hydroxycoumarin** | [C09315](http://www.genome.jp/dbget-bin/www_bget?cpd:C09315) |
| **Kynurenic acid** | [C01717](http://www.genome.jp/dbget-bin/www_bget?cpd:C01717) |
| **Palmitoylethanolamide** | No ID |
| **Sphingosine** | [C00319](http://www.genome.jp/dbget-bin/www_bget?cpd:C00319) |
| **Sphinganine** | [C00836](http://www.genome.jp/dbget-bin/www_bget?cpd:C00836) |
| Acyl carnitine (12:0) | No ID |
| **Ethyl arachidonate** | No ID |
| Acyl carnitine (13:1) | No ID |
| **Cholesterol** | [C00187](http://www.genome.jp/dbget-bin/www_bget?cpd:C00187) |
| Acyl carnitine (14:1) | No ID |
| Acyl carnitine (14:0) | No ID |
| **Riboflavin** | [C00255](http://www.genome.jp/dbget-bin/www_bget?cpd:C00255) |
| Acyl carnitine (15:0) | No ID |
| Acyl carnitine (16:1) | No ID |
| Palmitoylcarnitine | [C02990](http://www.genome.jp/dbget-bin/www_bget?cpd:C02990) |
| Acyl carnitine (17:1) | No ID |
| Acyl carnitine (18:2) | No ID |
| Acyl carnitine (18:1) | No ID |
| Acyl carnitine (18:0) | No ID |
| **α-Tocopherol** | [C02477](http://www.genome.jp/dbget-bin/www_bget?cpd:C02477) |
| Acyl carnitine 20:1) | No ID |
| Acyl carnitine (20:0) | No ID |
| **1-Myristoyl-glycero-3-phosphocholine** | No ID |
| Acyl carnitine (22:0) | No ID |
| **1-Palmitoyl-glycero-3-phosphocholine** | No ID |
| **1-Oleoyl-glycero-3-phosphocholine** | No ID |
| **1-Stearoyl-glycero-3-phosphocholine** | No ID |
| **1-Hexadecyl-2-acetyl-glycero-3-phosphocholine** | No ID |
| **Astaxanthin** | [C08580](http://www.genome.jp/dbget-bin/www_bget?cpd:C08580) |
| **Sphingomyelin(d18:1/16:0)** | No ID |

1) Kyoto Encyclopedia of Genes and Genomes (KEGG) (http://www.genome.jp/kegg/kegg2.html)

2) Chemical compound not annotated in animal metabolism map (bold). Some chemicals annotated in other organisms (bacteria, plants etc.) have KEGG ID.

3) Not identified chemical compounds found in some metabolome analysis
