# Supplementary material for: An aluminum shield enables the amphipod Hirondellea gigas to inhabit deep-sea environments
Source: PLoS One. 2019 Apr 4;14(4):e0206710. doi: 10.1371/journal.pone.0206710 (PMC6449124; doi:10.1371/journal.pone.0206710)
Supplement: S4 Table — (DOCX) [file pone.0206710.s016.docx]

S4 Table Amount of gluconic acid in the body of *H. gigas*

| Sample ID | Body fluid (mM) | | Adhered to Exoskeleton  (µ mol) |
| --- | --- | --- | --- |
|  | Water-phase | Lipid-phase |  |
| 1 | 0.34 ± 0.022 | N.D.^a)^ | 0.16 ± 0.08 |
| 2 | 0.43 ± 0.18 | N.D. | 0.063 ± 0.022 |
| 3 | 0.36 ± 0.07 | N.D. | 0.084 ± 0.034 |

a) Not detected
