# Supplementary material for: An aluminum shield enables the amphipod Hirondellea gigas to inhabit deep-sea environments
Source: PLoS One. 2019 Apr 4;14(4):e0206710. doi: 10.1371/journal.pone.0206710 (PMC6449124; doi:10.1371/journal.pone.0206710)
Supplement: S1 File — (DOCX) [file pone.0206710.s017.docx]

**Materials and Methods of supporting Figs and Tables**

**High-pressure experiment with salmon roe**

The salmon roe were purchased from a fish market. We washed the salmon roe with ice-cold artificial seawater (IWAKI CO., LTD, Tokyo, Japan) 3 times and then soaked them in the ice-cold artificial seawater. We created 16 tubes that each contained 4 salmon roe and artificial seawater, and we then added AlCl_3_ (final conc.: 10 mM) to half of the tubes and adjusted the pH to 8.0 with 5 M NaOH. The tubes were then pressurized at 100 MPa or 0.1 MPa and incubated at 2°C for 1 day. After decompression, we observed the salmon roe and measured the protein concentration of the artificial seawater using the Bradford method [1].

**Cloning of cytochrome oxidase gene from the coastal amphipods**

We extracted DNA from 10-20 coastal amphipods using DNAiso (Takara Bio Inc., Kyoto, Japan). The cytochrome oxidase I (COI) gene was amplified from the extracted DNA solution by PCR using the universal primer pair LCO1490 (5'-GGTCAACAAATCATAAAGATATTGG-3’) and HCO2198 (5'-TAAACTTCAGGGTGACCAAAAAATCA-3') [2]. PCR amplification with a 50-µl reaction volume was performed using the GeneAmp PCR System 9700 (Applied Biosystems, Carlsbad, CA, USA) with EmeraldAmp (Takara Bio Inc., Otsu, Japan) and the buffer supplied with the enzyme. The PCR conditions were as follows: an initial incubation at 96°C for 30 s, 25 cycles of 98°C for 30 s, an incubation at 55°C for 30 s, another incubation at 72°C for 1 min, and a final extension at 72°C for 5 min. The PCR products were cloned in pT7blue-2 vector (Merck Millipore, MA, USA) and then transformed into *Escherichia coli* DH5a for blue/white selection. The cloned COI gene was amplified from the white colony by PCR using the same primers and conditions. The PCR products were analyzed by electrophoresis on a 1% agarose gel. The gel was purified using Exo-SAP digestion with Exonuclease I (USB Corp., Cleveland, OH, USA) and shrimp alkaline phosphatase (SAP) (Promega, Fitchburg, WI, USA) at 37°C for 20 min and then treated at 80°C for 30 min to inactivate the enzymes. The PCR products were sequenced using the primers described above and DYEnamic ET Dye Terminator reagent (GE Healthcare Life Sciences, Piscataway, NJ, USA) on a MegaBACE 1000 (Amersham Biosciences, Piscataway, NJ, USA) automatic sequencer. The nucleotide sequences were trimmed, assembled, and translated using Sequencher 3.7 software (Gene Codes Corp., Ann Arbor, MI, USA).

**Phylogenetic analysis of the coastal amphipod**

A preliminary phylogenetic affiliation for each sequence was determined by conducting a BLAST search. The most closely related sequences with representative Lysianassoidean species sequences and certain outgroup sequences were aligned with our sequences using CLUSTALX, and ambiguous regions were excluded from the alignment. Phylogenetic trees were calculated with the PAML algorithm implemented in the TOPALi package ver. 2.5 [3]. The statistical robustness of the analysis was estimated by bootstrapping with 250 replicates.

**Metabolome analysis of *H. gigas***

A metabolome analysis was conducted from 1 frozen *H. gigas* individual using capillary electrophoresis time-of-flight mass spectrometry (CE-TOFMS) and liquid chromatograph time-of-flight mass spectrometry (LC-TOFMS). The metabolome measurements were conducted through the services of a facility at the Human Metabolome Technology Inc., Tsuruoka, Japan.

**CE-TOFMS**

Approximately 45 mg of a frozen individual was plunged into 1.5 ml of 50% acetonitrile/Milli-Q water containing internal standards (H3304-1002, Human Metabolome Technologies, Inc., Tsuruoka, Japan) at 0°C to inactivate the enzymes. The individual was homogenized three times at 1,500 rpm for 120 s using a tissue homogenizer (Shake Master neo, Bio Medical Science, Tokyo, Japan), and then the homogenate was centrifuged at 2,300 × *g* at 4°C for 5 min. Subsequently, 800 µL of the upper aqueous layer was centrifugally filtered through a Millipore 5 kDa cutoff filter at 9,100 × *g* at 4°C for 120 min to remove proteins. The filtrate was centrifugally concentrated and re-suspended in 50 µl of Milli-Q water for the CE-MS analysis.

A CE-TOFMS analysis was conducted using an Agilent CE Capillary Electrophoresis System equipped with an Agilent 6210 TOF mass spectrometer, Agilent 1100 Isocratic HPLC pump, Agilent G1603A CE-MS adapter kit, and Agilent G1607A CE-ESI-MS sprayer kit (Agilent Technologies, Waldbronn, Germany). The systems were controlled by the software Agilent G2201AA ChemStation version B.03.01 for CE (Agilent Technologies). The metabolites were analyzed using a fused-silica capillary (50

μm *i.d.*

×

80

cm total length) and a commercial electrophoresis buffer (Solution ID: H3301-1001 for the cation analysis and H3302-1021 for the anion analysis, Human Metabolome Technologies) as the electrolyte. The sample was injected at a pressure of 50

mbar for 10

 sec (approximately 10

nl) for the cation analysis and 25

sec (approximately 25

nl) for the anion analysis. The spectrometer was scanned from *m/z* 50 to 1,000. Other conditions were applied as previously described [4-6].

The automatic integration software MasterHands (Keio University, Tsuruoka, Japan) was used to obtain peak information, including the *m/z*, migration time for the CE-TOFMS measurement (MT) and the peak area [7]. Signal peaks corresponding to isotopomers, adduct ions, and other product ions of known metabolites were excluded, and the remaining peaks were annotated with putative metabolites from the HMT metabolite database based on their MTs and *m*/*z* values as determined by the TOFMS analysis. The tolerance range for the peak annotation was configured at ±0.5 min for MT and ±10 ppm for *m/z*. In addition, the peak areas were normalized against those of the internal standards, and the resultant relative area values were further normalized by the sample amount.

A hierarchical cluster analysis (HCA) and principal component analysis (PCA) were performed using our proprietary software PeakStat and SampleStat, respectively. The detected metabolites were plotted on metabolic pathway maps using VANTED (Visualization and Analysis of Networks containing Experimental Data) software [8].

**LC-TOFMS**

Approximately 45 mg of a frozen specimen was plunged into 0.5 ml of acetonitrile containing 1% formic acid and internal standards (H3304-1002, Human Metabolome Technologies, Inc., Tsuruoka, Japan) at 0°C to inactivate the enzymes. The specimen was homogenized three times at 1,500 rpm for 120 s using a tissue homogenizer (Shake Master neo), and then 167 µl of Milli-Q water was added and homogenized once at 1,500 rpm for 120 s. The homogenate was centrifuged at 5,000 × *g* at 4°C for 5 min. The supernatant was used as a sample for the LC-TOFMS analysis, and a precipitate was homogenized once with 0.667 ml in the same solution at 1,500 rpm for 120 s. The homogenate of the precipitate was centrifuged at 5,000 × *g* at 4°C for 5 min. Both supernatants were mixed and centrifugally filtered through a Nanosep 3K (PALL Co., NY, US) at 9,100 × *g* at 4°C for 120 min to remove proteins. Solid phase extraction was conducted on the filtrate to remove phospholipids. The filtrate was centrifugally concentrated and re-suspended in 100 µl of 50% (v/v) isopropanol solution for the LC-TOFMS analysis.

The LC-TOFMS analysis was conducted using an Agilent 1200 series RRLC system SL (Agilent Technologies, CA, USA) and an ODS column (2×50 mm, 2 µm) equipped with an Agilent LC/MSD TOF system (Agilent Technologies). The LC analysis was performed using a mobile phase of solution A (H_2_O/0.1%HCOOH) and solution B (isopropanol: acetonitrile: H_2_O (65: 30: 5)/0.1% HCOOH, 2 mM HCOONH_4_) at a gradient of 0-0.5 min: B 1%, 0.5-13.5 min: B 1-100%, 13.5-20 min: B 100%. Negative and positive modes were performed for the cationic and anionic metabolites. The MS system, measurement conditions, and analyses were conducted using the same procedures as described above for the CE-TOFMS analysis.

**Reference**

1. Bradford MM. A rapid and sensitive method for the quantitation of microgram quantities of protein utilizing the principle of protein- dye binding. Anal Biochem*.* 1976;72: 248–254.
2. Folmer O, Black M, Hoeh W, Lutz R, Vrijenhoek R. DNA primers for amplification of mitochondrial cytochrome c oxidase subunit I from diverse metazoan invertebrates. Mol Mar Biol Biotechnol. 1994;3: 294-299.
3. Milne I, Lindner D, Bayer M, Husmeier D, McGuire G, et al. TOPALi v2: a rich graphical interface for evolutionary analyses of multiple alignments on HPC clusters and multi-core desktops. Bioinformatics. 2009;25: 126-127.
4. Soga T, Heiger DN. Amino acid analysis by capillary electrophoresis electrospray ionization mass spectrometry. Anal.Chem. 2007;72: 1236–1241.
5. Soga T. Ueno Y, Naraoka H, Ohashi Y, Tomita M, et al. Simultaneous determination of anionic intermediates for *Bacillus subtilis* metabolic pathways by capillary electrophoresis electrospray ionization mass spectrometry. Anal Chem*.* 74;2002: 2233–2239.
6. Soga T. Ohashi Y, Ueno Y, Naraoka H, Tomita M, et al. Quantitative metabolome analysis using capillary electrophoresis mass spectrometry. J Proteome Res. 2003;2: 488–494.

S1 Table Amount of aluminum in the body of *H. gigas*

| Sample ID | Body fluid (µ mol) | | Exoskeleton  (µ mol) | Total  (µ mol) |
| --- | --- | --- | --- | --- |
|  | Water-phase | Lipid-phase (x10^-3^) |  |  |
| 1 | 0.255 ± 0.059 | 3.14 ± 1.7 | 1.05 ± 0.16 | 1.31 ± 0.22 |
| 2 | 0.827 ± 0.017 | 4.19 ± 0.32 | 1.29 ± 0.15 | 2.12 ± 0.17 |
| 3 | 1.16 ± 0.13 | 23.9 ± 6.8 | 1.18 ± 0.10 | 2.36 ± 0.24 |

S2 Table The TEM/EDS analysis of the sediments

|  | Sample ID | | | | | |
| --- | --- | --- | --- | --- | --- | --- |
| Element | #1 | #2 | #3 | #4 | #5 | #6 |
| C (standaed) | 66.07 | 54.12 | 72.49 | 63.84 | 74.13 | 71.41 |
| O | 6.55 | 9.51 | 4.57 | 8.52 | 5.07 | 6 |
| Mg | 2.13 | 3.24 | 1.34 | 2.02 | 1.99 | 0.77 |
| Al | 1.23 | 2.43 | 1.13 | 1.69 | 0.99 | 1.14 |
| Si | 5.6 | 8.72 | 3.77 | 6.71 | 3.6 | 5.66 |
| K | 0.15 | 0.3 | 0.2 | 0.18 | 0.19 | 0.33 |
| Fe | 2.44 | 2.99 | 1.93 | 3.7 | 0.97 | 2.5 |
| Cu^1)^ | 15.82 | 18.68 | 14.56 | 13.34 | 13.06 | 12.2 |
| Total | 99.99 | 99.99 | 99.99 | 100 | 100 | 100.01 |
| Al:Si | 4.55 | 3.59 | 3.34 | 3.97 | 3.64 | 4.96 |

Weight (%)

1) Background

S3 Table Metabolic analysis of *H. gigas*

| Compound name | KEGG ID^1)^ |
| --- | --- |
|  |  |
| Lactic acid | [C00186, C00256, C01432](http://www.genome.jp/dbget-bin/www_bget?cpd:C00186) |
| 2-Hydroxybutyric acid | [C05984](http://www.genome.jp/dbget-bin/www_bget?cpd:C05984) |
| Glyceric acid | [C00258](http://www.genome.jp/dbget-bin/www_bget?cpd:C00258) |
| Succinic acid | [C00042](http://www.genome.jp/dbget-bin/www_bget?cpd:C00042) |
| **2-Aminoethylphosphonic acid**^2)^ | [C03557](http://www.genome.jp/dbget-bin/www_bget?cpd:C03557) |
| **4-Methyl-2-oxovaleric acid 3-Methyl-2-oxovaleric acid** | [C00233 C00671, C03465](http://www.genome.jp/dbget-bin/www_bget?cpd:C00233) |
| Malic acid | [C00149, C00497, C00711](http://www.genome.jp/dbget-bin/www_bget?cpd:C00149) |
| **2-Hydroxyglutaric acid** | [C02630, C01087, C03196](http://www.genome.jp/dbget-bin/www_bget?cpd:C02630) |
| Xanthine | [C00385](http://www.genome.jp/dbget-bin/www_bget?cpd:C00385) |
| **Pelargonic acid** | [C01601](http://www.genome.jp/dbget-bin/www_bget?cpd:C01601) |
| Uric acid | [C00366](http://www.genome.jp/dbget-bin/www_bget?cpd:C00366) |
| Dihydroxyacetone phosphate | [C00111](http://www.genome.jp/dbget-bin/www_bget?cpd:C00111) |
| Glycerol 3-phosphate | [C00093](http://www.genome.jp/dbget-bin/www_bget?cpd:C00093) |
| 3-Phosphoglyceric acid | [C00197](http://www.genome.jp/dbget-bin/www_bget?cpd:C00197) |
| Citric acid | [C00158](http://www.genome.jp/dbget-bin/www_bget?cpd:C00158) |
| **Gluconic acid** | [C00257](http://www.genome.jp/dbget-bin/www_bget?cpd:C00257) |
| **Lauric acid** | [C02679](http://www.genome.jp/dbget-bin/www_bget?cpd:C02679) |
| Xanthurenic acid | [C02470](http://www.genome.jp/dbget-bin/www_bget?cpd:C02470) |
| Ribulose 5-phosphate | [C00199, C01101](http://www.genome.jp/dbget-bin/www_bget?cpd:C00199) |
| XA0033^3)^ | No ID |
| ***myo*-Inositol 1-phosphate *myo*-Inositol 3-phosphate** | [C01177 C04006](http://www.genome.jp/dbget-bin/www_bget?cpd:C01177) |
| Fructose 6-phosphate | [C05345, C00085](http://www.genome.jp/dbget-bin/www_bget?cpd:C05345) |
| Glucose 6-phosphate | [C00668, C01172, C00092](http://www.genome.jp/dbget-bin/www_bget?cpd:C00668) |
| Glucose 1-phosphate | [C00103](http://www.genome.jp/dbget-bin/www_bget?cpd:C00103) |
| Sedoheptulose 7-phosphate | [C05382](http://www.genome.jp/dbget-bin/www_bget?cpd:C05382) |
| 3'-CMP **2'-CMP** | [C05822 C03104](http://www.genome.jp/dbget-bin/www_bget?cpd:C05822) |
| cAMP | [C00575](http://www.genome.jp/dbget-bin/www_bget?cpd:C00575) |
| Fructose 1,6-diphosphate | [C00354](http://www.genome.jp/dbget-bin/www_bget?cpd:C00354) |
| AMP | [C00020](http://www.genome.jp/dbget-bin/www_bget?cpd:C00020) |
| **3'-AMP** | [C01367](http://www.genome.jp/dbget-bin/www_bget?cpd:C01367) |
| IMP | [C00130](http://www.genome.jp/dbget-bin/www_bget?cpd:C00130) |
| GMP | [C00144](http://www.genome.jp/dbget-bin/www_bget?cpd:C00144) |
| Trehalose 6-phosphate | [C00689](http://www.genome.jp/dbget-bin/www_bget?cpd:C00689) |
| ADP | [C00008](http://www.genome.jp/dbget-bin/www_bget?cpd:C00008) |
| ATP | [C00002](http://www.genome.jp/dbget-bin/www_bget?cpd:C00002) |
| Taurocholic acid | [C05122](http://www.genome.jp/dbget-bin/www_bget?cpd:C05122) |
| UDP-*N*-acetylglucosamine | [C00043](http://www.genome.jp/dbget-bin/www_bget?cpd:C00043) |
| NAD^+^ | [C00003](http://www.genome.jp/dbget-bin/www_bget?cpd:C00003) |
| Trimethylamine | [C00565](http://www.genome.jp/dbget-bin/www_bget?cpd:C00565) |
| **Ethanolamine** | [C00189](http://www.genome.jp/dbget-bin/www_bget?cpd:C00189) |
| Gly | [C00037](http://www.genome.jp/dbget-bin/www_bget?cpd:C00037) |
| β-Ala | [C00099](http://www.genome.jp/dbget-bin/www_bget?cpd:C00099) |
| Sarcosine | [C00213](http://www.genome.jp/dbget-bin/www_bget?cpd:C00213) |
| Ala | [C00041, C00133, C01401](http://www.genome.jp/dbget-bin/www_bget?cpd:C00041) |
| **Glycerol** | [C00116](http://www.genome.jp/dbget-bin/www_bget?cpd:C00116) |
| **Azetidine 2-carboxylic acid** | [C08267](http://www.genome.jp/dbget-bin/www_bget?cpd:C08267) |
| **3-Aminoisobutyric acid** | [C03284, C05145](http://www.genome.jp/dbget-bin/www_bget?cpd:C03284) |
| **2-Aminobutyric acid** | [C02261, C02356](http://www.genome.jp/dbget-bin/www_bget?cpd:C02261) |
| GABA | [C00334](http://www.genome.jp/dbget-bin/www_bget?cpd:C00334) |
| *N*,*N*-Dimethylglycine | [C01026](http://www.genome.jp/dbget-bin/www_bget?cpd:C01026) |
| 3-Aminobutyric acid | No ID |
| Choline | [C00114](http://www.genome.jp/dbget-bin/www_bget?cpd:C00114) |
| Ser | [C00065, C00716, C00740](http://www.genome.jp/dbget-bin/www_bget?cpd:C00065) |
| Hypotaurine | [C00519](http://www.genome.jp/dbget-bin/www_bget?cpd:C00519) |
| Histamine | [C00388](http://www.genome.jp/dbget-bin/www_bget?cpd:C00388) |
| Uracil | [C00106](http://www.genome.jp/dbget-bin/www_bget?cpd:C00106) |
| Creatinine | [C00791](http://www.genome.jp/dbget-bin/www_bget?cpd:C00791) |
| Pro | [C00148, C00763, C16435](http://www.genome.jp/dbget-bin/www_bget?cpd:C00148) |
| Betaine | [C00719](http://www.genome.jp/dbget-bin/www_bget?cpd:C00719) |
| Val | [C00183, C06417, C16436](http://www.genome.jp/dbget-bin/www_bget?cpd:C00183) |
| **5-Aminovaleric acid** | [C00431](http://www.genome.jp/dbget-bin/www_bget?cpd:C00431) |
| **2-Methylserine** | [C02115](http://www.genome.jp/dbget-bin/www_bget?cpd:C02115) |
| Thr | [C00188, C00820](http://www.genome.jp/dbget-bin/www_bget?cpd:C00188) |
| Cys | [C00097, C00736, C00793](http://www.genome.jp/dbget-bin/www_bget?cpd:C00097) |
| Nicotinic acid | [C00253](http://www.genome.jp/dbget-bin/www_bget?cpd:C00253) |
| Taurine | [C00245](http://www.genome.jp/dbget-bin/www_bget?cpd:C00245) |
| Thymine | [C00178](http://www.genome.jp/dbget-bin/www_bget?cpd:C00178) |
| Imidazole-4-acetic acid | [C02835](http://www.genome.jp/dbget-bin/www_bget?cpd:C02835) |
| XC0016^3)^ | No ID |
| ***N*-Methylproline** | No ID |
| *trans*-Glutaconic acid | [C02214](http://www.genome.jp/dbget-bin/www_bget?cpd:C02214) |
| *cis*-4-Hydroxyproline | [C03440](http://www.genome.jp/dbget-bin/www_bget?cpd:C03440) |
| Hydroxyproline | [C01157](http://www.genome.jp/dbget-bin/www_bget?cpd:C01157) |
| Creatine | [C00300](http://www.genome.jp/dbget-bin/www_bget?cpd:C00300) |
| Ile | [C00407, C06418, C16434](http://www.genome.jp/dbget-bin/www_bget?cpd:C00407) |
| Leu | [C00123, C01570, C16439](http://www.genome.jp/dbget-bin/www_bget?cpd:C00123) |
| **Norspermidine** | [C03375](http://www.genome.jp/dbget-bin/www_bget?cpd:C03375) |
| **Gly-Gly** | [C02037](http://www.genome.jp/dbget-bin/www_bget?cpd:C02037) |
| Asn | [C00152, C01905, C16438](http://www.genome.jp/dbget-bin/www_bget?cpd:C00152) |
| Ornithine | [C00077, C00515, C01602](http://www.genome.jp/dbget-bin/www_bget?cpd:C00077) |
| Thiaproline | No ID |
| Asp | [C00049, C00402, C16433](http://www.genome.jp/dbget-bin/www_bget?cpd:C00049) |
| Adenine | [C00147](http://www.genome.jp/dbget-bin/www_bget?cpd:C00147) |
| Hypoxanthine | [C00262](http://www.genome.jp/dbget-bin/www_bget?cpd:C00262) |
| Trigonelline | [C01004](http://www.genome.jp/dbget-bin/www_bget?cpd:C01004) |
| Stachydrine | [C10172](http://www.genome.jp/dbget-bin/www_bget?cpd:C10172) |
| γ-Butyrobetaine | [C01181](http://www.genome.jp/dbget-bin/www_bget?cpd:C01181) |
| Spermidine | [C00315](http://www.genome.jp/dbget-bin/www_bget?cpd:C00315) |
| Gln | [C00064, C00303, C00819](http://www.genome.jp/dbget-bin/www_bget?cpd:C00064) |
| Lys | [C00047, C00739, C16440](http://www.genome.jp/dbget-bin/www_bget?cpd:C00047) |
| *threo*-β-Methylaspartic acid | [C03618](http://www.genome.jp/dbget-bin/www_bget?cpd:C03618) |
| Glu | [C00025, C00217, C00302](http://www.genome.jp/dbget-bin/www_bget?cpd:C00025) |
| Met | [C00073, C00855, C01733](http://www.genome.jp/dbget-bin/www_bget?cpd:C00073) |
| Triethanolamine | [C06771](http://www.genome.jp/dbget-bin/www_bget?cpd:C06771) |
| Guanine | [C00242](http://www.genome.jp/dbget-bin/www_bget?cpd:C00242) |
| His | [C00135, C00768, C06419](http://www.genome.jp/dbget-bin/www_bget?cpd:C00135) |
| **Betonicine** | [C08269](http://www.genome.jp/dbget-bin/www_bget?cpd:C08269) |
| **Ala-Ala** | [C00993](http://www.genome.jp/dbget-bin/www_bget?cpd:C00993) |
| 2-Aminoadipic acid | [C00956](http://www.genome.jp/dbget-bin/www_bget?cpd:C00956) |
| Carnitine | [C00318, C00487, C15025](http://www.genome.jp/dbget-bin/www_bget?cpd:C00318) |
| Phe | [C00079, C02057, C02265](http://www.genome.jp/dbget-bin/www_bget?cpd:C00079) |
| Pyridoxamine | [C00534](http://www.genome.jp/dbget-bin/www_bget?cpd:C00534) |
| Tyr-Arg_divalent | No ID |
| 3-Methylhistidine | [C01152](http://www.genome.jp/dbget-bin/www_bget?cpd:C01152) |
| ***N*^5^-Ethylglutamine** | [C01047](http://www.genome.jp/dbget-bin/www_bget?cpd:C01047) |
| *N*-Acetylornithine | [C00437](http://www.genome.jp/dbget-bin/www_bget?cpd:C00437) |
| Citrulline | [C00327](http://www.genome.jp/dbget-bin/www_bget?cpd:C00327) |
| Arg | [C00062, C00792](http://www.genome.jp/dbget-bin/www_bget?cpd:C00062) |
| Glucosamine | [C00329](http://www.genome.jp/dbget-bin/www_bget?cpd:C00329) |
| Tyr | [C00082, C01536, C06420](http://www.genome.jp/dbget-bin/www_bget?cpd:C00082) |
| **Phosphorylcholine** | [C00588](http://www.genome.jp/dbget-bin/www_bget?cpd:C00588) |
| **Gly-Leu** | No ID |
| ***N*^6^-Acetyllysine** | [C02727](http://www.genome.jp/dbget-bin/www_bget?cpd:C02727) |
| *N*^6^,*N*^6^,*N*^6^-Trimethyllysine | [C03793](http://www.genome.jp/dbget-bin/www_bget?cpd:C03793) |
| **Homocitrulline** | [C02427](http://www.genome.jp/dbget-bin/www_bget?cpd:C02427) |
| **Gly-Asp** | No ID |
| **2,6-Diaminopimelic acid** | [C00666](http://www.genome.jp/dbget-bin/www_bget?cpd:C00666) |
| ***N*-Acetylhistidine** | [C02997](http://www.genome.jp/dbget-bin/www_bget?cpd:C02997) |
| **N,N-Dimethylarginine** | [C03626](http://www.genome.jp/dbget-bin/www_bget?cpd:C03626) |
| *O*-Acetylcarnitine | [C02571](http://www.genome.jp/dbget-bin/www_bget?cpd:C02571) |
| Trp | [C00078, C00525, C00806](http://www.genome.jp/dbget-bin/www_bget?cpd:C00078) |
| Kynurenine | [C00328, C01718](http://www.genome.jp/dbget-bin/www_bget?cpd:C00328) |
| **3-Methoxytyrosine** | No ID |
| XC0061^3)^ | No ID |
| **β-Ala-Lys** | [C05341](http://www.genome.jp/dbget-bin/www_bget?cpd:C05341) |
| ***N*-Acetylgalactosamine** | [C01132](http://www.genome.jp/dbget-bin/www_bget?cpd:C01132) |
| 3-Hydroxykynurenine | No ID |
| **γ-Glu-2-aminobutyric acid** | No ID |
| Ser-Glu | No ID |
| Cystine | [C00491, C01420](http://www.genome.jp/dbget-bin/www_bget?cpd:C00491) |
| Thymidine | [C00214](http://www.genome.jp/dbget-bin/www_bget?cpd:C00214) |
| Cytidine | [C00475](http://www.genome.jp/dbget-bin/www_bget?cpd:C00475) |
| Uridine | [C00299](http://www.genome.jp/dbget-bin/www_bget?cpd:C00299) |
| **Pyridoxamine 5'-phosphate** | [C00647](http://www.genome.jp/dbget-bin/www_bget?cpd:C00647) |
| γ-Glu-Cys | [C00669](http://www.genome.jp/dbget-bin/www_bget?cpd:C00669) |
| XC0089^3)^ | No ID |
| Glycerophosphocholine | [C00670](http://www.genome.jp/dbget-bin/www_bget?cpd:C00670) |
| Adenosine | [C00212](http://www.genome.jp/dbget-bin/www_bget?cpd:C00212) |
| 2'-Deoxyguanosine | [C00330](http://www.genome.jp/dbget-bin/www_bget?cpd:C00330) |
| Inosine | [C00294](http://www.genome.jp/dbget-bin/www_bget?cpd:C00294) |
| **Glu-Glu** | [C01425](http://www.genome.jp/dbget-bin/www_bget?cpd:C01425) |
| Saccharopine | [C00449](http://www.genome.jp/dbget-bin/www_bget?cpd:C00449) |
| Guanosine | [C00387](http://www.genome.jp/dbget-bin/www_bget?cpd:C00387) |
| **His-Glu** | No ID |
| Argininosuccinic acid | [C03406](http://www.genome.jp/dbget-bin/www_bget?cpd:C03406) |
| **Arg-Glu** | No ID |
| Glutathione (GSSG)_divalent | [C00127](http://www.genome.jp/dbget-bin/www_bget?cpd:C00127) |
| Glutathione (GSH) | [C00051](http://www.genome.jp/dbget-bin/www_bget?cpd:C00051) |
| Tyr-Glu | No ID |
| NMN | [C00455](http://www.genome.jp/dbget-bin/www_bget?cpd:C00455) |
| *S*-Adenosylhomocysteine | [C00021](http://www.genome.jp/dbget-bin/www_bget?cpd:C00021) |
| *S*-Adenosylmethionine | [C00019](http://www.genome.jp/dbget-bin/www_bget?cpd:C00019) |
| **Cysteine glutathione disulfide** | [C05526](http://www.genome.jp/dbget-bin/www_bget?cpd:C05526) |
| Myristic acid | [C06424](http://www.genome.jp/dbget-bin/www_bget?cpd:C06424) |
| Palmitoleic acid | [C08362](http://www.genome.jp/dbget-bin/www_bget?cpd:C08362) |
| Palmitic acid | [C00249](http://www.genome.jp/dbget-bin/www_bget?cpd:C00249) |
| Fatty acid (17:1) | No ID |
| Fatty acid (17:0) | No ID |
| Heptadecanoic acid | No ID |
| Stearidonic acid | [C16300](http://www.genome.jp/dbget-bin/www_bget?cpd:C16300) |
| Linolenic acid | [C06427](http://www.genome.jp/dbget-bin/www_bget?cpd:C06427) |
| Linoleic acid | [C01595](http://www.genome.jp/dbget-bin/www_bget?cpd:C01595) |
| Oleic acid | [C00712](http://www.genome.jp/dbget-bin/www_bget?cpd:C00712) |
| Stearic acid | [C01530](http://www.genome.jp/dbget-bin/www_bget?cpd:C01530) |
| Fatty acid (19:1) | No ID |
| *cis*-5,8,11,14,17-Eicosapentaenoic acid | [C06428](http://www.genome.jp/dbget-bin/www_bget?cpd:C06428) |
| Arachidonic acid | [C00219](http://www.genome.jp/dbget-bin/www_bget?cpd:C00219) |
| *cis*-8,11,14-Eicosatrienoic acid | [C03242](http://www.genome.jp/dbget-bin/www_bget?cpd:C03242) |
| *cis*-11,14-Eicosadienoic acid | [C16525](http://www.genome.jp/dbget-bin/www_bget?cpd:C16525) |
| ***cis*-11-Eicosenoic acid *cis*-13-Eicosenoic acid** | [C16526 No ID](http://www.genome.jp/dbget-bin/www_bget?cpd:C16526) |
| **15(S)-** **Hydroxyeicosatetraenoic acid** | [C04742](http://www.genome.jp/dbget-bin/www_bget?cpd:C04742) |
| *cis*-4,7,10,13,16,19-Docosahexaenoic acid | [C06429](http://www.genome.jp/dbget-bin/www_bget?cpd:C06429) |
| Fatty acid (22:5) | No ID |
| Fatty acid (22:4) | No ID |
| Fatty acid (22:2) | No ID |
| **Fatty acid (22:1) Erucic acid** | [No ID C08316](http://www.genome.jp/dbget-bin/www_bget?cpd:C08316) |
| **Prostaglandin D2** | [C00696](http://www.genome.jp/dbget-bin/www_bget?cpd:C00696) |
| **Prostaglandin D1** | [C06438](http://www.genome.jp/dbget-bin/www_bget?cpd:C06438) |
| Fatty acid (24:5) | No ID |
| Nervonic acid | [C08323](http://www.genome.jp/dbget-bin/www_bget?cpd:C08323) |
| **1-Palmitoyl-glycero-3-phosphoethanolamine** | No ID |
| **7-Hydroxycoumarin** | [C09315](http://www.genome.jp/dbget-bin/www_bget?cpd:C09315) |
| **Kynurenic acid** | [C01717](http://www.genome.jp/dbget-bin/www_bget?cpd:C01717) |
| **Palmitoylethanolamide** | No ID |
| **Sphingosine** | [C00319](http://www.genome.jp/dbget-bin/www_bget?cpd:C00319) |
| **Sphinganine** | [C00836](http://www.genome.jp/dbget-bin/www_bget?cpd:C00836) |
| Acyl carnitine (12:0) | No ID |
| **Ethyl arachidonate** | No ID |
| Acyl carnitine (13:1) | No ID |
| **Cholesterol** | [C00187](http://www.genome.jp/dbget-bin/www_bget?cpd:C00187) |
| Acyl carnitine (14:1) | No ID |
| Acyl carnitine (14:0) | No ID |
| **Riboflavin** | [C00255](http://www.genome.jp/dbget-bin/www_bget?cpd:C00255) |
| Acyl carnitine (15:0) | No ID |
| Acyl carnitine (16:1) | No ID |
| Palmitoylcarnitine | [C02990](http://www.genome.jp/dbget-bin/www_bget?cpd:C02990) |
| Acyl carnitine (17:1) | No ID |
| Acyl carnitine (18:2) | No ID |
| Acyl carnitine (18:1) | No ID |
| Acyl carnitine (18:0) | No ID |
| **α-Tocopherol** | [C02477](http://www.genome.jp/dbget-bin/www_bget?cpd:C02477) |
| Acyl carnitine 20:1) | No ID |
| Acyl carnitine (20:0) | No ID |
| **1-Myristoyl-glycero-3-phosphocholine** | No ID |
| Acyl carnitine (22:0) | No ID |
| **1-Palmitoyl-glycero-3-phosphocholine** | No ID |
| **1-Oleoyl-glycero-3-phosphocholine** | No ID |
| **1-Stearoyl-glycero-3-phosphocholine** | No ID |
| **1-Hexadecyl-2-acetyl-glycero-3-phosphocholine** | No ID |
| **Astaxanthin** | [C08580](http://www.genome.jp/dbget-bin/www_bget?cpd:C08580) |
| **Sphingomyelin(d18:1/16:0)** | No ID |

1) Kyoto Encyclopedia of Genes and Genomes (KEGG) (http://www.genome.jp/kegg/kegg2.html)

2) Chemical compound not annotated in animal metabolism map (bold). Some chemicals annotated in other organisms (bacteria, plants etc.) have KEGG ID.

3) Not identified chemical compounds found in some metabolome analysis

S4 Table Amount of gluconic acid in the body of *H. gigas*

| Sample ID | Body fluid (mM) | | Adhered to Exoskeleton  (µ mol) |
| --- | --- | --- | --- |
|  | Water-phase | Lipid-phase |  |
| 1 | 0.34 ± 0.022 | N.D.^a)^ | 0.16 ± 0.08 |
| 2 | 0.43 ± 0.18 | N.D. | 0.063 ± 0.022 |
| 3 | 0.36 ± 0.07 | N.D. | 0.084 ± 0.034 |

a) Not detected
